# Pea PSII-LHCII supercomplexes form pairs by making connections across the stromal gap

**DOI:** 10.1038/s41598-017-10700-8

**Published:** 2017-08-30

**Authors:** Pascal Albanese, Roberto Melero, Benjamin D Engel, Alessandro Grinzato, Paola Berto, Marcello Manfredi, Angelica Chiodoni, Javier Vargas, Carlos Óscar Sánchez Sorzano, Emilio Marengo, Guido Saracco, Giuseppe Zanotti, Jose-Maria Carazo, Cristina Pagliano

**Affiliations:** 1 Applied Science and Technology Department–BioSolar Lab, Politecnico di Torino, Viale T. Michel 5, 15121 Alessandria, Italy; 2 Department of Biology, University of Padova, Via Ugo Bassi 58 B, 35121 Padova, Italy; 3 Biocomputing Unit, Centro Nacional de Biotecnología–CSIC, Darwin 3, Cantoblanco, 28049 Madrid, Spain; 4 Department of Molecular Structural Biology, Max Planck Institute of Biochemistry, 82152 Martinsried, Germany; 5 Department of Biomedical Sciences, University of Padova, Via Ugo Bassi 58 B, 35121 Padova, Italy; 6 ISALIT–Department of Science and Technological Innovation, University of Eastern Piedmont, Viale T. Michel 11, 15121 Alessandria, Italy; 7 Department of Science and Technological Innovation, University of Eastern Piedmont, Viale T. Michel 11, 15121 Alessandria, Italy; 8 Center for Sustainable Future Technologies – CSFT@POLITO, Istituto Italiano di Tecnologia, Corso Trento 21, 10129 Torino, Italy

## Abstract

In higher plant thylakoids, the heterogeneous distribution of photosynthetic protein complexes is a determinant for the formation of grana, stacks of membrane discs that are densely populated with Photosystem II (PSII) and its light harvesting complex (LHCII). PSII associates with LHCII to form the PSII-LHCII supercomplex, a crucial component for solar energy conversion. Here, we report a biochemical, structural and functional characterization of pairs of PSII-LHCII supercomplexes, which were isolated under physiologically-relevant cation concentrations. Using single-particle cryo-electron microscopy, we determined the three-dimensional structure of paired C_2_S_2_M PSII-LHCII supercomplexes at 14 Å resolution. The two supercomplexes interact on their stromal sides through a specific overlap between apposing LHCII trimers and via physical connections that span the stromal gap, one of which is likely formed by interactions between the N-terminal loops of two Lhcb4 monomeric LHCII subunits. Fast chlorophyll fluorescence induction analysis showed that paired PSII-LHCII supercomplexes are energetically coupled. Molecular dynamics simulations revealed that additional flexible physical connections may form between the apposing LHCII trimers of paired PSII-LHCII supercomplexes in appressed thylakoid membranes. Our findings provide new insights into how interactions between pairs of PSII-LHCII supercomplexes can link adjacent thylakoids to mediate the stacking of grana membranes.

## Introduction

Photosystem II (PSII) is a multisubunit pigment-protein complex that is embedded in the thylakoid membranes of oxygenic photosynthetic organisms. It uses solar energy to catalyse the splitting of water into dioxygen, protons and electrons, thus providing the molecular oxygen and chemical energy that sustain most of the life on Earth^1^.

PSII forms supercomplexes that are composed of two moieties: a central PSII core containing the catalytic reaction center and a peripheral antenna system responsible for harvesting light and transferring energy to the reaction center. The PSII core, occurring mainly in dimeric form^2–4^, is highly conserved among all oxygenic photosynthetic organisms, with the exception of the extrinsic proteins of the oxygen evolving complex (PsbO, PsbV and PsbU in cyanobacteria and diatoms; PsbO, PsbP and PsbQ in plants and green algae)^5^. Conversely, the peripheral antenna system is evolutionarily divergent; it is composed of extrinsic phycobilisomes in cyanobacteria, while it consists of intrinsic LHCII light harvesting complexes in green algae and plants^5, 6^. The evolution of different antenna systems was accompanied by the differentiation of thylakoid membranes into functionally distinct appressed (stacked) and non-appressed (unstacked) regions, which in higher plants are called grana and stroma lamellae, respectively^7^.

Two related processes are believed to govern grana stacking: (i) interactions between adjacent membrane surfaces^8^ and (ii) lateral segregation of photosystems within the thylakoid membrane plane^9, 10^. At neutral pH, thylakoid membranes carry a net negative charge, and the maintenance of the grana stacks requires shielding cations (e.g., Mg^2+^, K^+^ and Na^+^)^11, 12^. Indeed, it has long been known that exposure of thylakoid membranes to low-ionic strength buffers induces grana unstacking, and the readdition of low concentrations of divalent cations (≥ 5 mM Mg^2+^) or higher concentrations of monovalent cations (≥ 150 mM K^+^ or Na^+^) results in spontaneous restacking^11^. Photosynthetic membrane proteins are segregated into different thylakoid domains; grana regions contain mainly PSII and LHCII, complexes with flat stromal surfaces that do not project into the narrow stromal gap between thylakoids. Conversely, stroma lamellae and grana end membranes accommodate Photosystem I (PSI), with its light harvesting antenna complex (LHCI), and ATP synthase (ATP-ase), which have bulky stromal protrusions. Unlike the photosystems, cytochrome *b*_6_ */f* (Cyt *b*_6_ */f*) is evenly distributed between stacked and unstacked thylakoid regions^13, 14^. This lateral heterogeneity between PSI and PSII is thought to be intimately linked to membrane appression, allowing higher plants to optimize photosynthesis in ever-changing light conditions^15^.

Grana formation seems to be driven by interactions between the stromal domains of LHCIIs that face each other from adjacent stacked membranes^16, 17^. However, grana with apparently normal stacked architecture form in *Arabidopsis* mutants that are virtually devoid of LHCII^18^, suggesting that other mechanisms are involved in grana formation^19^. In addition, it has been proposed that the ordered assembly of semi-crystalline 2D arrays of PSII-LHCII supercomplexes in grana thylakoids might promote grana stacking by mediating specific contacts between adjacent membrane discs^20^. While the lateral heterogeneity of thylakoid membrane proteins appears to drive grana staking, there seems to be a reciprocal relationship where thylakoid architecture enforces this lateral heterogeneity; the unstacking of grana with low-ionic strength buffer causes the random redistribution of photosystems within the lateral thylakoid membrane plane as well as the detachment of some LHCII from supercomplexes^21, 22^.

In plant grana thylakoids, the PSII core associates with a variable number of LHCII antennas to form different types of PSII-LHCII supercomplexes^14, 23, 24^. The C_2_S_2_ supercomplex consists of a dimeric PSII core (C_2_), which strongly binds two LHCII trimers (S-trimers) via two copies of the monomeric Lhcb4 and Lhcb5 subunits. Larger C_2_S_2_M_1-2_ supercomplexes contain two copies of monomeric Lhcb6, with one or two additional LHCII trimers (M-trimers) bound with moderate strength to the dimeric PSII core via Lhcb4 and Lhcb6^14^.

Two 3D maps of the C_2_S_2_ PSII-LHCII supercomplex isolated from spinach have been obtained by single-particle cryo-electron microscopy (cryo-EM); the first structure was generated 17 years ago at 17 Å resolution^25^, while the second structure was recently solved at 3.2 Å resolution^26^. The new structure’s significant improvement in resolution allowed the precise positioning of the pigments bound to the PSII-LHCII supercomplex. Thus, there is now a clear structural description for the energy transfer from the peripheral LHCII antennas to the PSII reaction center. The next step for the mechanistic understanding of plant photosynthesis is to expand this structural description to the organization and coordination of multiple PSII-LHCII supercomplexes within the grana stack. Here, we pursue this goal with a structural and functional description of paired C_2_S_2_M PSII-LHCII supercomplexes that can link adjacent grana membranes.

## Results and Discussion

### Isolation of paired PSII-LHCII supercomplexes

The differentiation of thylakoid membranes into grana and stroma lamellae is a ubiquitous feature of higher plant chloroplasts^7^. Even though the number and diameter of discs within grana stacks may vary depending on fluctuating environmental conditions^27–29^, different plant species grown under the same illumination regime can differ intrinsically in their grana organization^30^. We used pea plants as starting material for PSII-LHCII supercomplex isolation because their chloroplasts contain large, highly stacked grana^30^.

In order to investigate how PSII-LHCII supercomplexes interact with each other within grana stacks, thylakoids were isolated from pea leaves in the presence of divalent cation concentrations (i.e., 5 mM Mg^2+^) that resemble the native chloroplast ionic environment^31^, preserving the stacked morphology of the grana membranes. PSII-LHCII supercomplexes were subsequently isolated by a quick (1 min) direct solubilization of stacked thylakoids with the mild detergent n-dodecyl-α-D-maltoside (α-DDM) followed by sucrose density gradient ultracentrifugation in the presence of the same concentration of divalent cations, according to an optimized protocol previously described^32^. Physiological cation concentrations were maintained throughout the entire purification, and either Ca^2+^ or Mg^2+^ cations in the sucrose gradient buffer produced the same isolation profile (data not shown). It is important to point out that our isolation procedure greatly differs from that used by Wei *et al*.^26^ to produce the recent cryo-EM structure of the unpaired C_2_S_2_ plant supercomplex. In their study, grana membranes were first isolated by solubilizing thylakoids with Triton X-100 and then unstacked by washing with a metal chelating agent (i.e., EDTA), followed by further solubilization with α-DDM and ultracentrifugation. The buffers were devoid of salt throughout the purification, and thus the resulting structure lacks the native interactions between supercomplexes found within stacked grana.

### Cryo-EM structure of paired C_2_S_2_M supercomplexes at 14 Å resolution

To investigate the structure of the isolated PSII-LHCII supercomplexes, the detergent-solubilized PSII-LHCII particles were frozen in vitreous ice and then imaged by transmission electron microscopy using a direct electron detector. From 6,834 micrographs collected (Supplementary Fig. 1a), 33,729 particles were manually picked for further data processing. After several image sorting steps and iterative rounds of 2D classification (Supplementary Fig. 1b), 6,776 particles corresponding to contaminants and blurred or broken complexes were discarded. The initial model (Supplementary Fig. 1c), built from 44 representative 2D classes, was used for the 3D classification of 26,953 particles, resulting in three classes: paired C_2_S_2_M supercomplexes (~53%), unpaired C_2_S_2_M supercomplexes (~21%) and paired C_2_S_2_ supercomplexes (~26%) (Supplementary Fig. 1d). From this classification, the majority of the isolated particles were found to be in a paired conformation (~79%), with the C_2_S_2_M form (~72%) more abundant than the C_2_S_2_ (~28%). Most particles contained LHCII M-trimers (~74%), irrespective of their paired or unpaired behaviour. This finding agrees with previous results showing that C_2_S_2_M is the predominant supercomplex in the thylakoid membranes of pea plants grown in moderate light intensity^33^, the same light condition used in this study.

**Figure 1:**
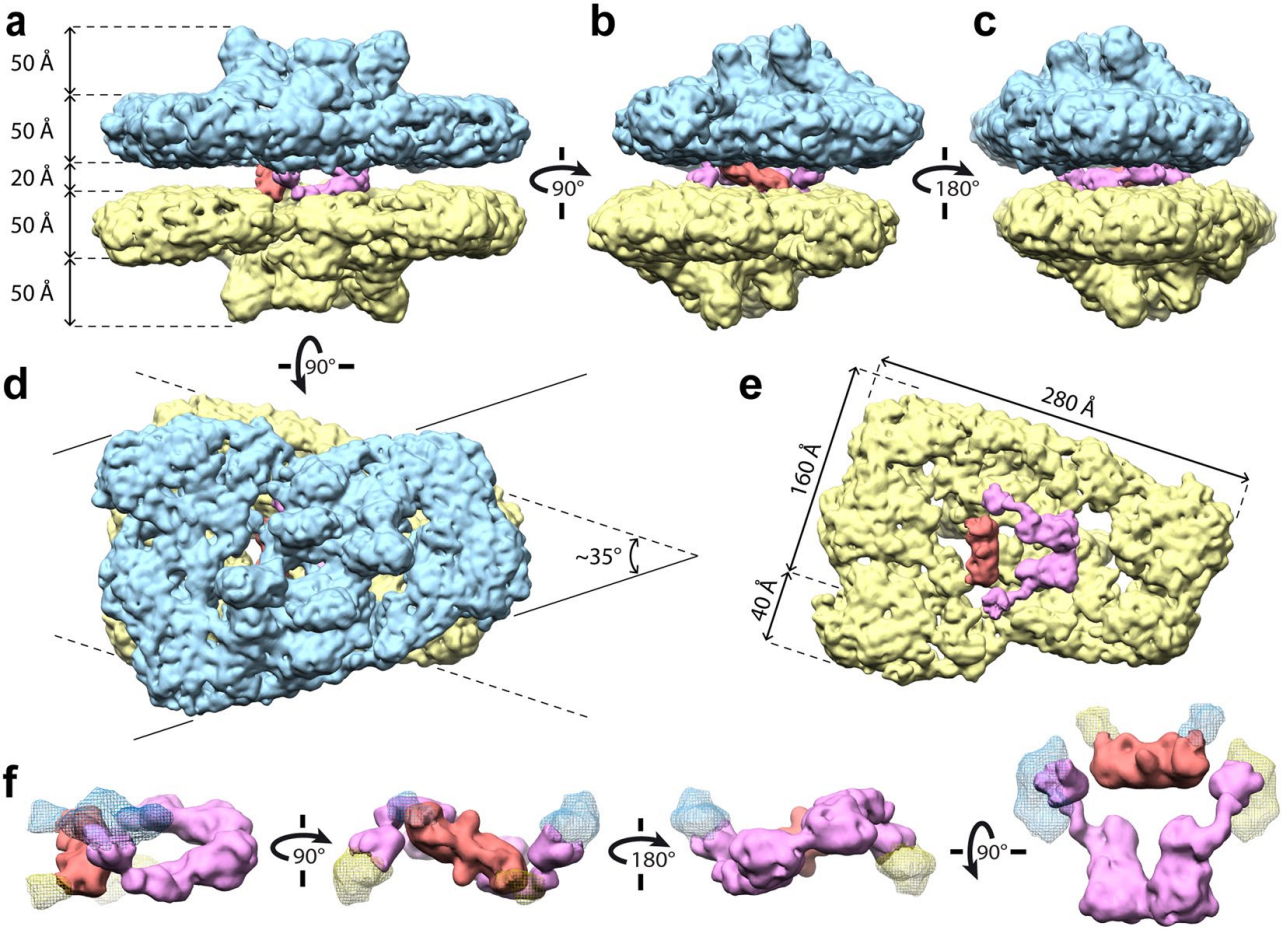
Three-dimensional cryo-EM density map of paired C_2_S_2_M PSII-LHCII supercomplexes. (**a**–**c**) Side view (**a**) and end views (**b**,**c**) within the membrane plane. (**d**) Top view towards the luminal surface of the paired supercomplexes, rotated 90° from panel (**a**). Solid and dashed lines extending from the top (cyan) and bottom (yellow) supercomplexes, respectively, illustrate the ~35^°^ rotational offset between the supercomplexes. (**e**) The same view as panel (**d**) with the top supercomplex removed, revealing the top view of the lower supercomplex’s stromal surface from the perspective of the stromal gap. The stromal connections are also displayed. (**f**) Structural features of the “hinge” (magenta) and “knot” (red) stromal connections shown in different orientations starting on the left with the same side view as panel (**a**). Contact sites to the upper and lower supercomplexes are indicated with cyan and yellow mesh densities, respectively.

Paired C_2_S_2_M particles, representing the most abundant 3D class and the largest isolated supercomplexes, were further analysed (Supplementary Fig. 1e,f). Several refinement cycles led to a final 3D cryo-EM map at an overall resolution of 14 Å (Fig. 1a–d), as judged by the “gold standard” Fourier shell correlation (FSC = 0.143) criterion^34^ (Supplementary Fig. 2). The 3D map depicts two C_2_S_2_M supercomplexes with their stromal surfaces facing each other. The supercomplexes do not completely overlap, but rather have a rotational offset of ~35° around the membrane plane’s normal vector (Fig. 1d). The 3D map of each C_2_S_2_M supercomplex, including the detergent shell, is 280 Å along its long-axis in the membrane plane, 200 Å along the M-trimer side and 160 Å along the S-trimer side (Fig. 1e). The thickness of the supercomplex is ~50 Å at its periphery, while the central protrusions extend for another ~50 Å on the luminal side (Fig. 1a). The central regions of the paired supercomplexes are connected on their stromal sides by two distinct densities that we have termed the “hinge” and “knot” as a description of their shapes (Fig. 1e,f). The ~20 Å stromal distance between the paired supercomplexes is consistent with reported stromal gap values for grana thylakoids, which range from 2 nm^14^ to 3.2–3.6 nm^20, 35^. In total, the supercomplex pair has a thickness of 220 Å, which is compatible with the *in situ* stacking periodicity measured for intact pea chloroplasts^20^.

### Model fitting reveals different degrees of overlap between facing LHCII S- and M-trimers

**Figure 2:**
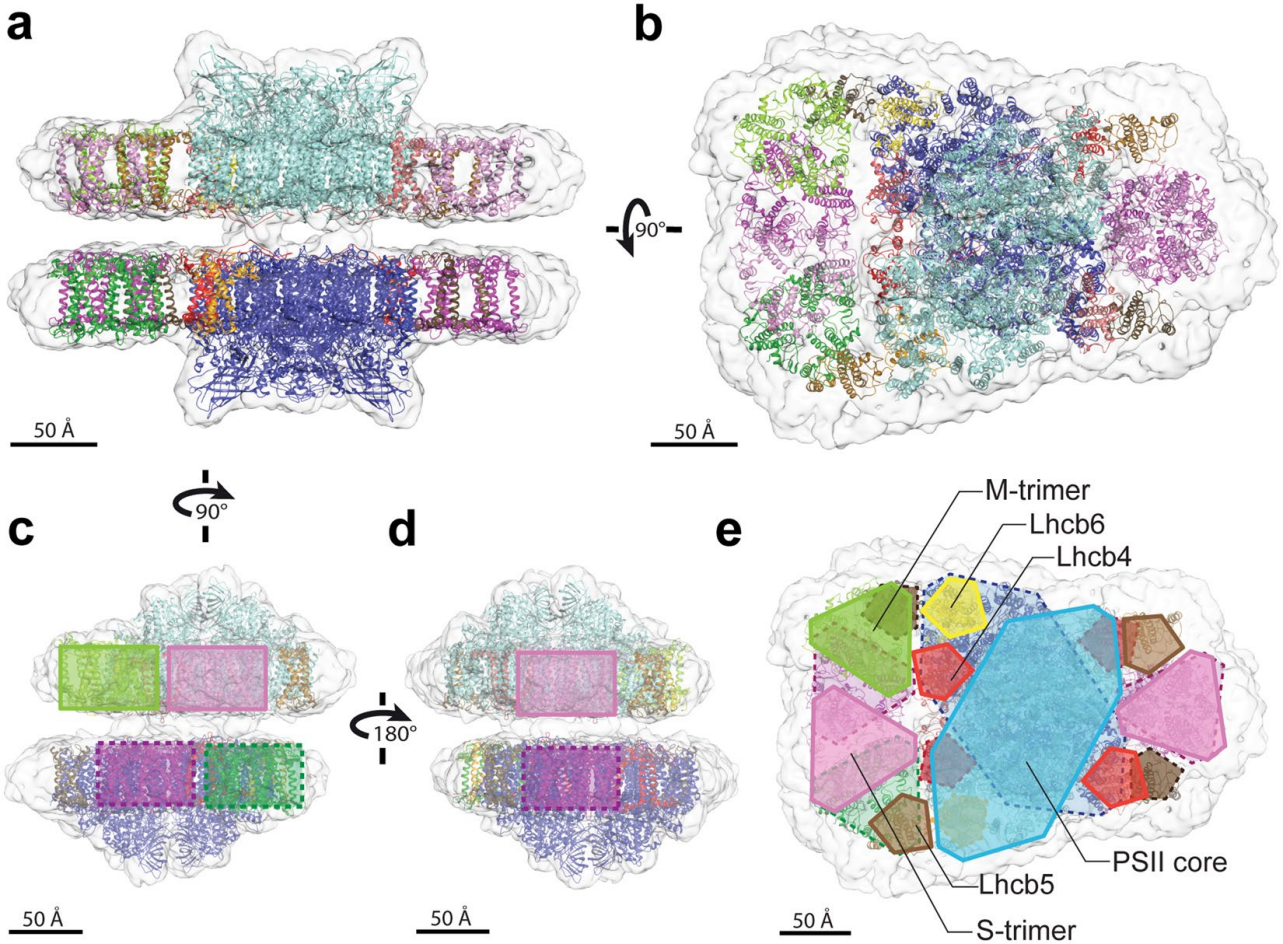
Fitting the cryo-EM density map of paired C_2_S_2_M supercomplexes with high-resolution structures. Side view within the membrane plane (**a**) and top view towards the luminal surface (**b**) of the paired C_2_S_2_M supercomplexes. The following structures were placed into the cryo-EM map by rigid-body fitting: the PSII dimeric core and monomeric Lhcb4 and Lhcb5 from spinach^26^ (PDB: 3JCU depleted of subunits PsbP, PsbQ, PsbTn; upper PSII dimer in cyan, lower PSII dimer in blue, upper Lhcb4 in pale red, lower Lhcb4 in dark red, upper Lhcb5 in pale brown, lower Lhcb5 in dark brown), the LHCII trimer from pea^36^ (PDB: 2BHW; upper S-trimers in pink, lower S-trimers in violet, upper M-trimer in pale green, lower M-trimer in dark green), the predicted structure of monomeric Lhcb6 from pea generated by the PHYRE2 algorithm^39^ (upper Lhcb6 in yellow, lower Lhcb6 in orange). (**c**–**e**) Schematic representations showing the positions of the LHCII trimers (**c**,**d**) and of all fitted supercomplex components (**e**), superimposed on the cryo-EM density map, showing end views within the membrane plane (**c**,**d**) and a top view towards the luminal surface (**e**). Colors match the structures in panels (**a**,**b**); solid lines for upper supercomplex components, dashed lines for lower supercomplex components.

To interpret our 3D cryo-EM map, we performed rigid-body fitting using the atomic coordinates from two structures: the 3.2 Å cryo-EM structure of the unpaired C_2_S_2_ PSII-LHCII supercomplex from spinach^26^ (PDB ID: 3JCU) for modeling the central dimeric PSII core together with the monomeric Lhcb4 and Lhcb5 subunits, and the 2.5 Å crystal structure of the LHCII trimer from pea^36^ (PDB ID: 2BHW) for modeling the LHCII S- and M-trimers. The atomic coordinates of the PSII dimeric core, the LHCII trimers, and the monomeric Lhcb4 and Lhcb5 fit well into the cryo-EM density map (Fig. 2). While modeling the PSII dimeric core, we did not fit the extrinsic subunits PsbP, PsbQ and PsbTn, since previous biochemical and proteomic characterization showed that PsbQ and PsbTn are absent from the supercomplexes and PsbP is present in sub-stoichiometric amounts relative to the core subunits^32, 37^. Although a higher-resolution crystal structure of spinach Lhcb4 (2.8 Å) is available^38^, it lacks 73 amino acid residues at the N-terminus, which were nearly all detected in our sample by mass spectrometry (Supplementary Table 1). Thus, we decided to fit our cryo-EM map with the more complete structure of Lhcb4 obtained by Wei *et al*.^26^. An atomic structure is not yet available for the monomeric Lhcb6 antenna, which links the LHCII M-trimer to the CP47 subunit of the PSII core. To model Lhcb6 within our cryo-EM map, we used the 3D structure predicted by the PHYRE2 program^39^ for the *P*. *sativum* Lhcb6 protein sequence, which showed 50% coverage in the mass spectrometry analysis of our sample (see Supplementary Table 1).

Despite the limited resolution of our 3D map, fitting high-resolution components of the PSII-LHCII supercomplex clearly showed that LHCII trimers face each other across the stromal gap with various degrees of overlap. On the side of the paired supercomplexes containing only S-trimers, the apposing LHCII trimers completely overlapped and were in close proximity to the core complex, with the same position observed in the structure by Wei *et al*.^26^ (Fig. 2b,d). Conversely, on the opposite side of the paired supercomplexes containing additional M-trimers, the S-trimers were more distantly positioned with respect to the core complex if compared to the structure by Wei *et al*.^26^, and the apposing S- and M-trimers only partially overlapped (Fig. 2b,c). The overlaps observed between pairs of facing LHCII trimers, including S- with S- and S- with M- trimers, were specific and closely resembled two arrangements observed in the crystal structure of multilayer packed LHCII from spinach reported by Wan *et al*.^40^. The crystal packing of LHCII in this study, obtained with a physiologically-relevant cation concentration and in the presence of native membrane lipids, may replicate the stacking of LHCII in native grana membranes. In these crystals, cations are sandwiched between the neighbouring LHCII layers to screen the surface charges and mediate interactions by forming strong salt bridges between highly conserved negatively charged residues on facing LHCII trimers. Although we did not resolve physical contacts between apposing LHCII trimers in our cryo-EM map (Fig. 1a), the conservation of these negatively charged residues in the Lhcb1 and Lhcb2 subunits was confirmed in our preparation (Supplementary Fig. 3a, Supplementary Table 1). This evidence provides a plausible explanation for the complete overlap observed between two S-trimers (Fig. 2b,d), which are composed of Lhcb1 and Lhcb2^3^, and thus have affinity for each other across the stromal gap due to salt bridge formation. It also helps explain the partial overlap between S- and M-trimers on the other side of the paired supercomplexes (Fig. 2b,c); the M-trimer contains Lhcb3^41^, which may lack affinity for other LHCII subunits, thus reducing trimer overlap on that side.

Our cryo-EM map provides clear evidence at intermediate resolution for interactions between apposing LHCII trimers within paired PSII-LHCII supercomplexes. In so doing, it bridges the resolution gap between previous reports of LHCII interactions from X-ray crystallography of isolated LHCII trimers^40^ and cryo-electron tomography of C_2_S_2_ supercomplex arrays embedded within intact grana membranes^20^.

### “Hinge” and “knot” densities make physical connections across the stromal gap

Well-defined electron densities were visible in the stromal gap between the paired supercomplexes, strongly indicating the presence of two different centrally positioned physical connections with distinct shapes and specific connections to the two C_2_S_2_M supercomplexes.

**Figure 3:**
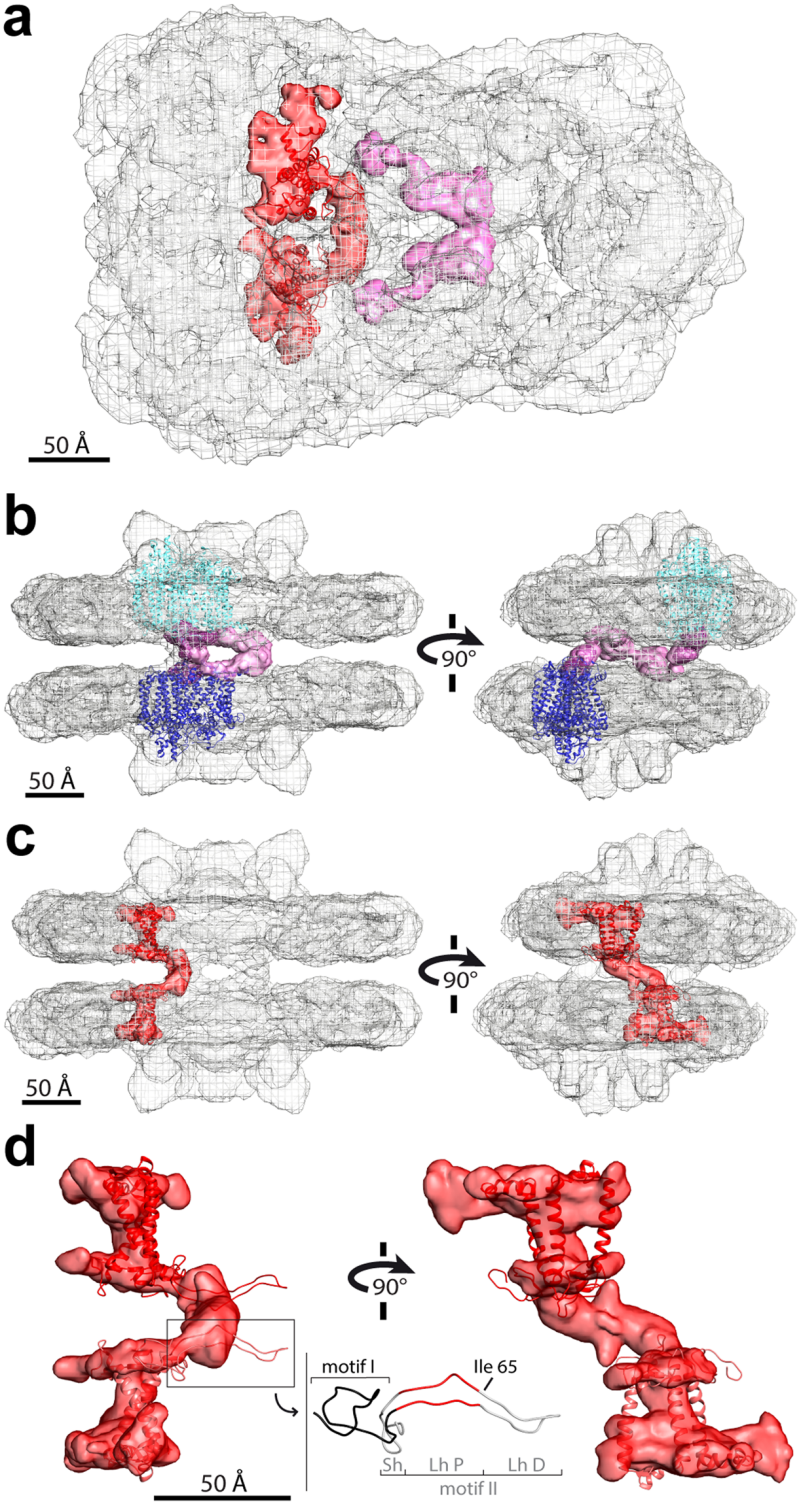
“Hinge” and “knot” connections cross the stromal gap of paired C_2_S_2_M supercomplexes. **(a)** Top view towards the luminal surface, showing the cryo-EM density map of paired C_2_S_2_M supercomplexes (grey mesh), with the stromal “hinge” density in magenta and the stromal “knot” density plus adjoining supercomplex region in red. Fitting of the high-resolution structure of Lhcb4 from spinach^26^ (PDB 3JCU: chain r; upper Lhcb4 in lighter red, lower Lhcb4 in darker red) as in Fig. 2 is shown. (**b**) Side and end views within the membrane plane showing the connections between the paired supercomplexes and the “hinge” (connecting supercomplex density in violet mesh). The hinge connects one PSII monomer of the upper supercomplex to one PSII monomer of the lower supercomplex (PDB 3JCU: chains A, C and D, corresponding to the subunits D1, CP43 and D2; upper PSII core in cyan, lower PSII core in blue)^26^. (**c)** Side and end views within the membrane plane showing the connections between the paired supercomplexes and the “knot”, which links an Lhcb4 of the upper supercomplex with an Lhcb4 of the lower supercomplex. Fitting of the high-resolution structure of Lhcb4 from spinach^26^ as in panel (**a**). (**d**) Enlarged views of the “knot” density plus adjoining supercomplex region fit with two Lhcb4 subunits, as in panel (**c**). The inset shows the N-terminal loop of the Lhcb4 structure by Wei *et al*.^26^, composed of motif I (Pro12–Lys41) in black and motif II (Pro42–Phe87) in grey and red. Motif II is subdivided into the short hairpin (Sh, Ala73–Phe87, grey), the proximal part of the long hairpin that fits within the “knot” cryo-EM density (Lh P, Pro42–Gln47 and Ile66–Ser72, red) and the distal part of the long hairpin that protrudes from the “knot” density (Lh D, Thr48–Ile65, grey). The flexible Ile65 residue, which separates the proximal and distal segments of the long hairpin, is indicated.

Towards the side of the paired supercomplexes containing only S-trimers, we observed a dimer of bulky densities with a “hinge” shape, which made defined contacts to PSII core subunits in each of the facing supercomplexes (Figs 1f and 3a,b). Comparison to the Wei *et al*.^26^ structure showed that this “hinge” density connects one PSII monomer in the upper supercomplex to one PSII monomer in the lower supercomplex, binding areas containing loops of the D1, CP43 and D2 proteins (Fig. 3b). Extensive biochemical and proteomic characterization of the sample used in this cryo-EM study^32, 37^ revealed the presence of an additional subunit of the PSII core that was not found in the Wei *et al*.^26^ unpaired C_2_S_2_ structure, a 10 kDa protein named PsbR. To date, the structure of PsbR is unknown and its positioning is still controversial. Although an extrinsic luminal location was hypothesized for PsbR based on its capacity to crosslink with the luminal PsbP subunit, PsbR’s hydrophobic C-terminus was suggested to be responsible for anchoring PsbR to the thylakoid membrane, leading to the protein’s insolubility^42^. Studies with *Arabidopsis psbR* knockout mutants revealed a reduction of the amount of PsbP bound to the PSII core and indicated a role for PsbR in stabilizing the supramolecular organization of PSII-LHCII supercomplexes^43^. However, it is not yet clear whether PsbR provides a direct docking site for PsbP or rather if it indirectly enhances the binding of PsbP on the luminal side by inducing a structural rearrangement in the PSII core. Taking all of this evidence into account, a possible interpretation of the “hinge” density in our cryo-EM map is that it is an extrinsic protein, potentially corresponding to PsbR. This protein, interacting with the stromal loops of the PSII core proteins, may induce a structural reorganization of PSII on the luminal side that favors the binding of PsbP, which stabilizes the association of LHCIIs with PSII within the membrane plane. In addition, by making connections that span the stromal gap between facing supercomplexes, the “hinge” can facilitate the stacking of adjacent grana thylakoids.

Towards the side of the paired supercomplexes containing the M-trimers, we observed a defined stromal density with a “knot” shape, forming an arm that extends diagonally across the stromal gap and contacts the supercomplexes on the opposite sides from the “hinge” binding sites (Figs 1f and 3a,c,d). This density is characterized by a symmetric and well-structured appearance, even when no D2 point group symmetry is imposed during image processing (data not shown). From its position in our fitted cryo-EM map, it is evident that the “knot” connection arises from the stromal surfaces of the Lhcb4 subunits, and thus can be assigned with high confidence to the N-terminal loops of two Lhcb4 subunits that face each other from two apposing supercomplexes (Fig. 3c,d). Lhcb4 contains a flexible ~90 amino acid N-terminal loop, easily lost during the crystallization process^38^, with dimensions that support its possible involvement in forming the “knot”. According to the structure by Wei *et al*.^26^, the N-terminal loop of Lhcb4 forms two motifs with irregular coil structures: motif I (Pro12–Lys41) and motif II (Pro42–Phe87) (Fig. 3d). Motif I forms a short loop near the stromal surface. Motif II contains a short hairpin loop (Ala73–Phe87) and a second hairpin loop that is approximately 40 Å long (Pro42–Ser72), extending into the stromal gap and then running nearly parallel to the stromal surface. In our structure, the connecting “knot” density that we attributed to the N-terminal loop of Lhcb4 confidently accommodates the proximal part of the motif II long hairpin formed approximately by Pro42–Gln47 and Ile66–Ser72. However, the distal part of the hairpin, corresponding approximately to Thr48–Ile65, protrudes from our 3D map (Fig. 3c,d). Spectroscopic analyses^44^ revealed that Ile65 is highly mobile, likely serving as a point of flexibility in the loop, while the Thr48–Ile65 region contains a helical stretch and a random coil. It is therefore plausible that the distal part of the long hairpin is responsible for “tying the knot” by homodimerizing to form the structured area at the center of the connecting density between the two Lhcb4 subunits.

The stromal gap-spanning connection between apposing Lhcb4 subunits may be secured by cation-mediated ionic interactions through the formation of salt bridges. Although the N-terminus of Lhcb4 contains some positively-charged amino acids, it is characterized by an overall net negative charge (Supplementary Fig. 3b). In particular, the distal part of the motif II long hairpin (Thr48–Ile65) is enriched in negatively-charged amino acids (Supplementary Fig. 3b) that are highly conserved in higher plants (Supplementary Fig. 4). Cations may screen these surface charges, forming strong salt bridges between negatively charged residues that mediate the interaction between the ends of two facing hairpin loops. Conversely, the proximal Ile66–Ser72 portion of the long hairpin as well as the short hairpin (Ala73–Phe87) each contain a Thr residue that becomes phosphorylated under high-light conditions^45^, indicating that these regions may regulate the homo-interactions of Lhcb4. Taken together, these results suggest that the Lhcb4 N-terminal loop may assume multiple conformations. Upon environmental light changes, phosphorylation of Lhcb4 might induce a conformational change in its N-terminal loop that “unties the knot”, allowing the redistribution of Lhcb4 from PSII-LHCII supercomplexes to PSII dimers and monomers^45^, while potentially enabling dynamic modulation of PSII-LHCII supercomplex pairing and ultimately the degree of grana staking.

### Cations mediate the physical connection between paired PSII-LHCII supercomplexes

**Figure 4:**
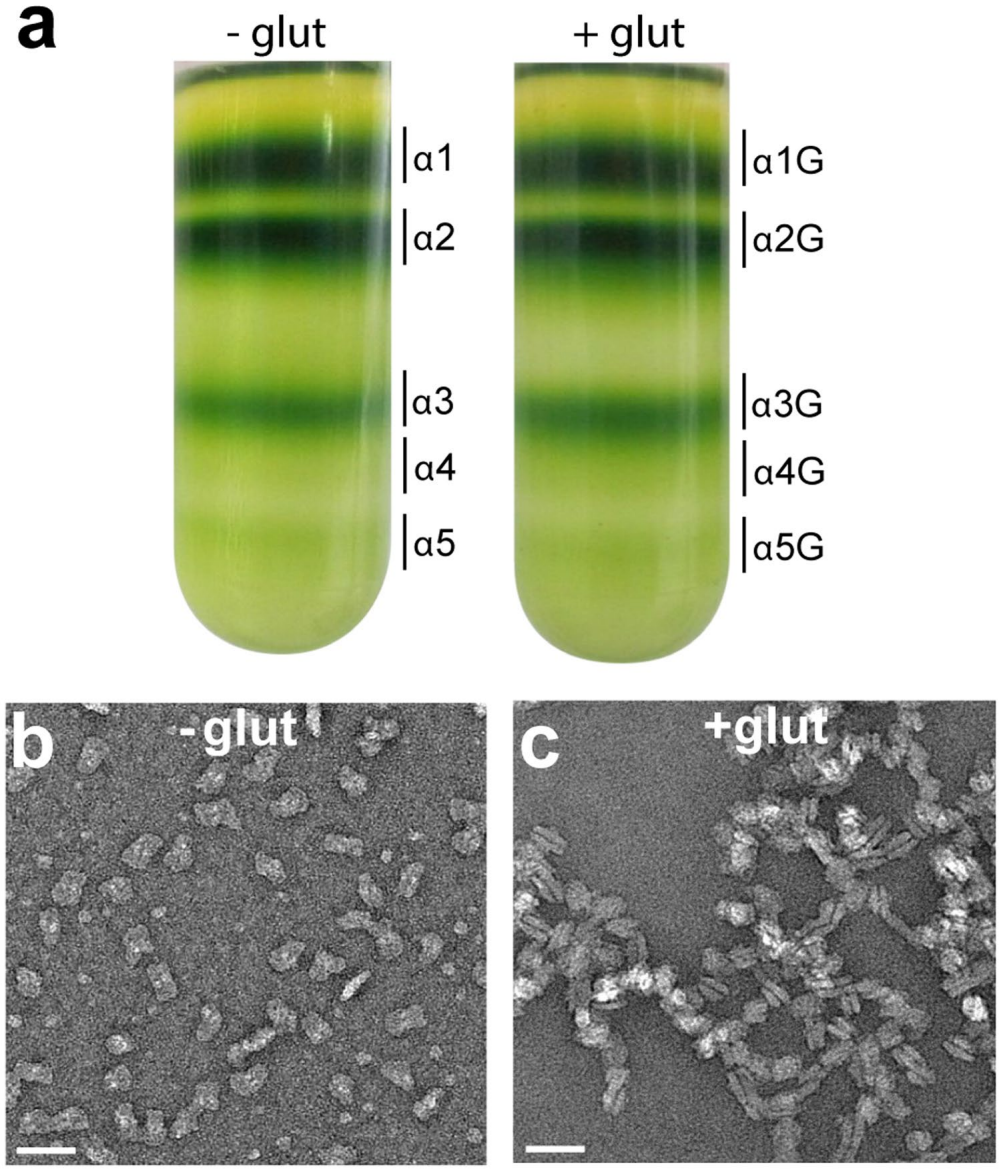
Salt removal causes unpairing of purified pea PSII-LHCII supercomplexes. **(a)** Isolation of PSII-LHCII supercomplexes from stacked pea thylakoid membranes by sucrose density gradients either containing ( + glut) or lacking glutaraldehyde (−glut). (**b**,**c)** Electron micrographs of uranyl acetate-stained PSII-LHCII supercomplexes contained in sucrose gradient bands α3 (**b**) and α3G (**c**) upon salt removal. Scale bar, 50 nm.

Despite the well-established requirement of shielding cations for the maintenance of grana stacking^11, 12^, the role of cations in PSII organization within grana remains unclear. Therefore, we assessed the potential function of cations in mediating the pairing of isolated PSII-LHCII supercomplexes. First, we isolated PSII-LHCII supercomplexes with sucrose density gradients that either contained (+glut) or lacked (-glut) glutaraldehyde, a cross-linking reagent often used to obtain information on protein interactions and the oligomerization state of complexes^46, 47^. Similar density gradient profiles of solubilized thylakoids were obtained for both conditions (Fig. 4a). PSII-LHCII supercomplexes, present in bands α3 and α3G, migrated to the same position on the sucrose gradient (Fig. 4a) and showed similar spectroscopic properties (Supplementary Fig. 5a). EM analysis, in cryo conditions for the α3 particles (Supplementary Fig. 1a) and by negative stain for the α3G particles (Supplementary Fig. 5b), showed that both samples contained paired supercomplexes. Next, buffer exchange was used to remove salt from both the + glut and −glut samples. Negative stain EM revealed that the majority of the supercomplexes detached from their paired partner upon salt removal (Fig. 4b), while the glutaraldehyde fixation was effective in maintaining the stromal connections between paired supercomplexes (Fig. 4c). Native polyacrylamide gel electrophoresis (PAGE) of the isolated supercomplexes (Supplementary Fig. 5c) confirmed that + glut particles (α3G) were in a paired oligomerization state, whereas -glut particles (α3) were unpaired. These results strongly support the hypothesis that the stromal connections between apposing supercomplexes are mediated by ionic bonds, which can be easily lost by changing the ionic strength of the buffers used during the isolation procedure. Notably, previous studies that reported the purification of paired PSII-LHCII supercomplexes also used cations in their isolation buffers^2, 25^. Recently, we observed a higher-order PSII-LHCII supercomplex oligomerization state consisting of two PSII-LHCII supercomplexes sitting side-by-side in the membrane plane (i.e., megacomplex), interacting with another two side-by-side supercomplexes across the stromal gap^48^. These paired megacomplexes correspond to band α5 of the sucrose gradient shown in Fig. 4a. Native PAGE showed that the removal of cations disrupted the stromal connections between facing megacomplexes (α5) unless these megacomplexes had been crosslinked with glutaraldehyde (α5G) (Supplementary Fig. 5c). In contrast, the lateral interactions between two supercomplexes were maintained without crosslinking, suggesting a specific role of salts in mediating the stromal connections between supercomplexes in adjacent thylakoid membranes.

### Paired PSII-LHCII supercomplexes are energetically coupled

Having assessed the requirement of cations for maintaining the structural connections between paired supercomplexes, we next sought to understand the function of supercomplex pairing by analyzing the fast chlorophyll *a* fluorescence induction curve^49^, which can be used to provide an estimation of the excitonic connectivity between PSII units^50^.

The fast phase of the chlorophyll *a* fluorescence induction curve is known as the OJIP transient, where O corresponds to the minimal fluorescence F_0_, J and I are inflections, and P is the peak corresponding to the maximal fluorescence F_m._ Three rise phases of the F_0_ to F_m_ kinetics are distinguishable: O–J (0–2 ms), J–I (2–30 ms), and I-P (30–1000 ms)^49^. In the analysis of the OJIP transient, the major inflection points of the fast fluorescence induction curve are used for the calculation of several parameters characterizing the structure and photochemical activity of a photosynthetic sample^50^.

**Figure 5:**
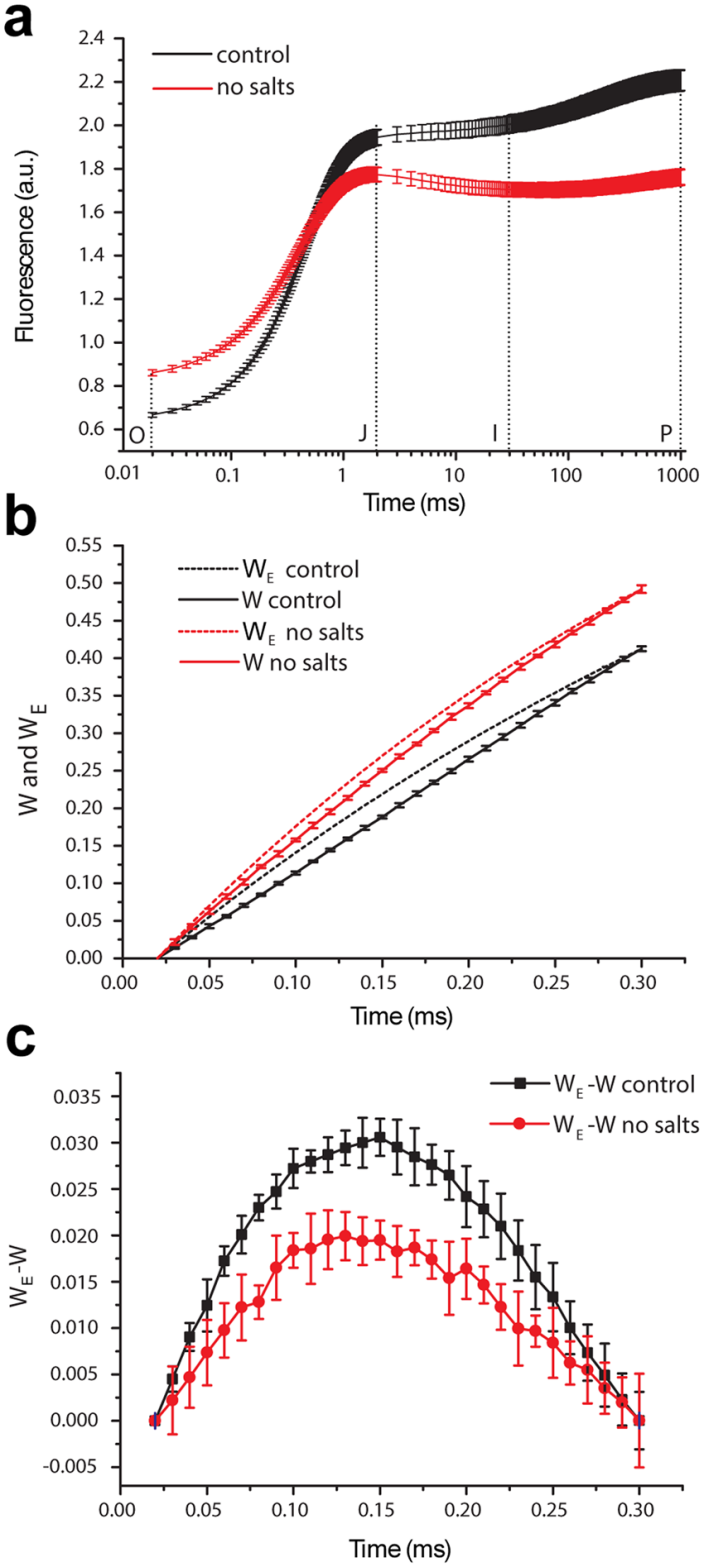
Estimation of the PSII excitonic connectivity in paired PSII-LHCII supercomplexes. (**a**) Chlorophyll *a* fluorescence induction curves (i.e., the OJIP transient) plotted on a logarithmic time scale (up to 1 s) for the paired PSII-LHCII supercomplexes prepared at 1.25 mg mL^−1^ in the sucrose gradient buffer and diluted 1,000-fold either in the sucrose gradient buffer (control, black line) or in a similar salt-free buffer (no salts, red line). The excitation light intensity was 2,400 μmol photons m^−2^ s^−1^, with an emission peak at 630 nm. (**b**) The initial 300 µs from the OJIP transient in panel (**a**) replotted on a linear time scale. The normalized O–J phase of the OJIP curve is labeled W (solid line), and the theoretical exponential curve corresponding to the unconnected system is labeled W_E_ (dashed line). For calculation of W and W_E_ see Supplementary Table 2. (**c**) Plot of W_E_ – W using the values from panel (**b**). Graphs display the mean values ± standard deviations of six replicates.

OJIP curves were recorded for isolated PSII-LHCII supercomplexes that had been diluted 1,000-fold either in the standard sucrose gradient buffer containing divalent cations or in a similar buffer that lacked salts (Fig. 5a). Compared to supercomplex pairs, unpaired supercomplexes in salt-free buffer showed increased values of F_0_ and reduced values of F_m_, with a concomitant strong reduction of the OJIP curve. The differences in OJIP curve shape and F_0_/F_m_ ratio may reflect different redox states of the plastoquinone Q_A_ and differences in macromolecular organization. In particular, the rise of the F_0_ under low ionic conditions might be due to the disconnection of some LHCII from the supercomplexes, as suggested by negative stain EM that showed the detachment of LHCII trimers from PSII-LHCII particles upon salt removal (Fig. 4b).

Since the shape of the induction curve is influenced by excitation energy transfer between PSII units, commonly referred to as PSII connectivity^51^ or grouping^52^, the sigmoidicity of the initial phase of the fast fluorescence transient can be used to estimate the degree of PSII connectivity^51^. To estimate the energetic PSII connectivity in paired and unpaired PSII-LHCII supercomplexes, we evaluated the O–J phase of the OJIP transient using the method developed by Strasser and Stirbet^53^. In this approach, the degree of the PSII connectivity can be measured and compared between different samples by estimating the sigmoidicity of the chlorophyll *a* fluorescence induction curve during the first few microseconds. For the first 300 µs of the OJIP curves recorded in Fig. 5a, we calculated the normalized O–J phase (labelled W) and the theoretical exponential curve corresponding to an unconnected system (labelled W_E_, for definitions and calculation of these parameters see Supplementary Table 2). The increase in sigmoidicity from the theoretical unconnected curve W_E_ to the experimental curve W was greater for the cation-containing sample than for the sample in salt-free buffer (Fig. 5b). The greater increase in sigmoidicity for the paired PSII-LHCII supercomplexes was further emphasized by plotting the difference between W_E_ and W (Fig. 5c).

The O–J phase of the OJIP curve was also used to estimate the parameters of connectivity between PSII units^49–53^ (for definitions see Supplementary Table 2). The O–J curvature parameter *C* and the connectivity among PSII units parameter *p* were over two times higher in paired PSII-LHCII supercomplexes (*C* ≈ 0.35, *p* ≈ 0.30) than in unpaired supercomplexes treated with salt-free buffer (*C* ≈ 0.14, *p* ≈ 0.13) (Table 1). In addition, the overall grouping probability *p2G* and the probability of connectivity between PSII units *ω* (for definitions see Supplementary Table 2), indicated a significantly higher connectivity in paired PSII-LHCII supercomplexes (*p2G* ≈ 0.21, *ω* ≈ 0.20) than in unpaired supercomplexes (*p2G* ≈ 0.15, *ω* ≈ 0.06) (Table 1).

**Table 1:** Selected parameters derived from fast fluorescence kinetic measurements to estimate the energetic connectivity between PSII units. Parameters for the paired PSII-LHCII supercomplexes prepared at 1.25 mg mL^−1^ in the sucrose gradient buffer and diluted 1,000-fold either in the sucrose gradient buffer (control) or in a similar salt-free buffer (no salts) were calculated according to methods previously reported^49–53^ as defined in Supplementary Table 2.

	*C*	*p*_*2G*_	*p*	*ω*
*control*	0.35 ± 0.03	0.21 ± 0.02	0.30 ± 0.02	0.20 ± 0.01
*no salts*	0.14 ± 0.03	0.15 ± 0.03	0.13 ± 0.02	0.06 ± 0.01

Despite the limitations of calculations based on OJIP curve measurements, these results support the conclusion that excitation energy migrates between supercomplex pairs that interact across the stromal gap. This suggests that energetic coupling is possible between two reaction center cores that are not members of the same PSII dimer. However, at the limited resolution of our cryo-EM map, we cannot deduce the precise route for this excitation energy diffusion. A plausible hypothesis is that energy crosses the ~20 Å stromal gap via interactions between overlapping LHCII trimers, which may be precisely positioned to face each other by the more robust “knot” and “hinge” physical connections.

### Molecular dynamics simulations predict additional transient interactions between overlapping LHCII trimers

**Figure 6:**
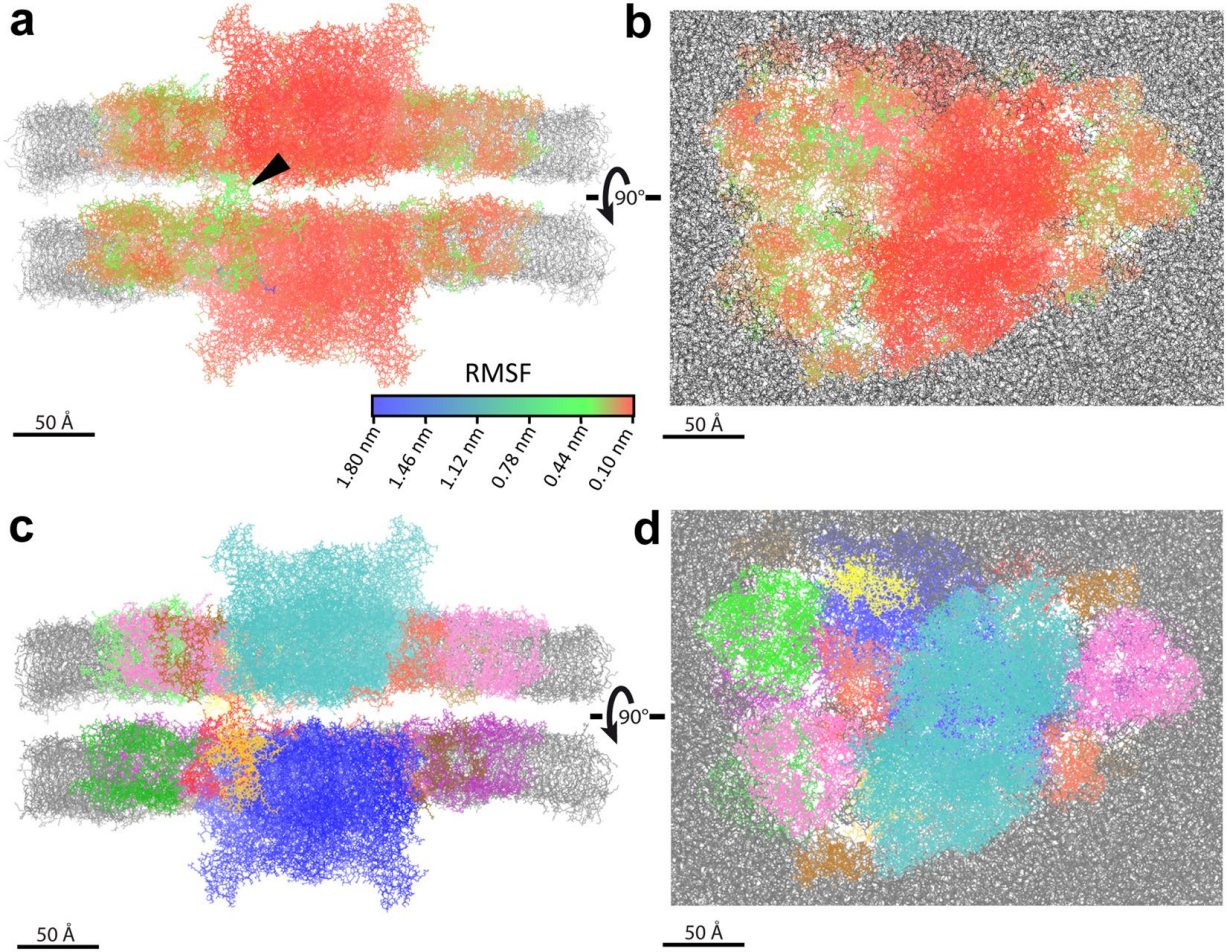
Molecular dynamics simulations. **(a**,**b)** Root mean square fluctuations (RMSF) for the backbone atoms of the paired C_2_S_2_M PSII-LHCII supercomplexes at the end of the 7 ns molecular dynamics simulation. Supercomplexes are colored according to RMSF values, and thylakoid membranes are grey. Side view within the membrane plane (**a**) and top view towards the luminal surface (**b**) of the paired C_2_S_2_M supercomplexes, with interactions that match the position of the “knot” connection in the stromal gap indicated with a black triangle. (**c**,**d**) Corresponding side view (**c**) and top view (**d**) of the paired C_2_S_2_M supercomplexes colored by subunit identity as in the Supplementary Video and Fig. 2.

To further investigate the interactions of paired C_2_S_2_M PSII-LHCII supercomplexes across the stromal gap, we ran molecular dynamics simulations using an atomic model generated from the fit of our cryo-EM density map (Fig. 2). After running the simulation for 7 ns, the backbone atoms had an average root mean square fluctuation (RMSF) of 0.23 nm compared to the reference time-averaged structure, with the central PSII core showing higher rigidity than the peripheral LHCII antennae system (Fig. 6 and Supplementary Fig. 6). Within several ps of starting the simulation, visible connections formed between the two apposing supercomplexes embedded within the appressed thylakoid membranes. Although the amino acid residues that form these connections varied during the simulation, they belong to the LHCII trimers and the monomeric Lhcb subunits (see Supplementary Video). These results suggest that flexible physical connections might occur between the antenna systems of PSII-LHCII supercomplexes that face each other across the stromal gap. The stromal N-terminal loops of Lhcb4 subunits positioned at the “knot” density seen in our cryo-EM map immediately formed connections that lasted throughout the entire timeframe of the simulation; conversely, the connections observed between the overlapping LHCII trimers were transient (see Supplementary Video). No strong connections formed at the position of the “hinge” density seen in the cryo-EM map, in agreement with the hypothesis that the proteins comprising this structure are not present in our fitted atomic model. The results of these molecular dynamics simulations are consistent with our cryo-EM study and may explain why only the most durable connections are well resolved in our cryo-EM map of paired C_2_S_2_M supercomplexes (Fig. 3).

## Conclusions

The structure reported here for paired C_2_S_2_M PSII-LHCII supercomplexes is representative of the predominant form of PSII in pea plants grown at moderate light intensity. The interaction observed between apposing supercomplexes is mediated by physical connections that span the stromal gap, one of which is likely provided by the mutual interaction of two Lhcb4 subunits, possibly through salt bridges between negatively-charged amino acids in their N-termini. In addition, there is a specific overlap between facing LHCII trimers that may be due to the formation of salt bridges between highly conserved negatively-charged residues of the Lhcb subunits in facing LHCII trimers. The preservation of the PSII-LHCII supercomplex paired architecture requires the maintenance of physiological cation concentrations in all the buffers used during the purification procedure. This suggests that cation salt bridges mediate both the physical connections across the stromal gap as well as the transient interactions between facing LHCII trimers.

Migration of excitation energy occurs between supercomplexes that interact across the stromal gap. Molecular dynamics simulations of the paired PSII-LHCII supercomplexes suggest different roles for the physical connections and the overlapping LHCII trimers; the former likely serve a structural function to hold the supercomplexes together, whereas the latter may enable energy transfer between PSII cores embedded in adjacent thylakoid membranes. Thus, the stromal gap-spanning interactions between PSII-LHCII supercomplexes likely enable both grana stacking and efficient energy transfer between adjacent thylakoids.

It remains to be tested how the physical connections and energetic coupling we observed in isolated paired C_2_S_2_M supercomplexes compare to PSII-LHCII supercomplexes within intact thylakoid membranes. Exploration of this interplay between supercomplex organization and thylakoid architecture will benefit from an extensive visualization of supercomplexes within the native thylakoid membranes using *in situ* cryo-electron tomography^54^. Until now, evidences of contacts between PSII-LHCII supercomplexes occurring in adjacent thylakoid membranes were provided by cryo-electron tomography^20^, revealing interactions mediated by the stromal surfaces of both the LHCIIs and the PSII reaction center dimers. In this study, the geometry observed for the interaction of supercomplexes in two adjacent membranes was different from that seen in our study, but this may be due to the specific arrangement in 2D arrays of the supercomplexes observed in the tomograms. Moreover, *Arabidopsis* mutants respond to a lack of conventional LHCII trimers by generating Lhcb5-containing trimers^18, 55^, and to a lack of PSII cores by accumulating large amounts of LHCII organized into three dimensional structures^56^ to ultimately maintain their grana architecture. Therefore, the functional interactions we characterized for purified pairs of C_2_S_2_M PSII-LHCII supercomplexes are likely one of the several possible mechanisms occurring *in vivo* within adjacent thylakoid membranes that plants adopted to secure a functional grana stacking among different flexible strategies evolved to thrive in ever-changing light conditions.

## Methods

### Plant growth and isolation of thylakoid membranes

Pea plants (*Pisum sativum* L., var. Palladio nano) were grown in a growth chamber (SANYO MLR-351H) at 20 °C and 60% humidity, with a 8 h light/16 h dark photoperiod under 150 µmol photons m^−2^ s^−1^.

Thylakoid membranes were isolated from three-week-old leaves using the following procedure that maintains the stacked granal organization. These samples are hereafter referred to as stacked thylakoid membranes. Pea leaves were disrupted by grinding with a blender in 50 mM HEPES pH 7.5, 300 mM sucrose and 5 mM MgCl_2_. The suspension was filtered through four cotton cloth layers, and the filtrate was centrifuged at 1,500 *g* for 10 min. The pellet was washed once by centrifugation in the same buffer and then homogenized in 5 mM MgCl_2_ and diluted 1:1 with 50 mM MES pH 6.0, 400 mM sucrose, 15 mM NaCl and 5 mM MgCl_2_, followed by 10 min centrifugation at 3,000 *g*. The resulting pellet of stacked thylakoid membranes was washed once by centrifugation in 25 mM MES pH 6.0, 10 mM NaCl and 5 mM MgCl_2_, and then suspended and stored in 25 mM MES pH 6.0, 10 mM NaCl, 5 mM MgCl_2_ and 2 M glycine betaine. When necessary, stacked thylakoids were flash frozen in liquid nitrogen and stored at −80 °C.

### Purification of pea PSII-LHCII supercomplexes

PSII-LHCII supercomplexes were isolated according to a previously optimized protocol^32^. Briefly, stacked thylakoid membranes at a Chl concentration of 1 mg mL^−1^ were treated with 50 mM n-dodecyl-α-D-maltoside (α-DDM) for 1 min at 4 °C in the dark. Phenylmethylsulphonylfluoride (500 mM) was present during the solubilization to inhibit protease activity. After centrifuging at 21,000 *g* for 10 min at 4 °C, 700 µl of the supernatant were added to the top of a linear sucrose gradient, prepared by freezing and thawing ultracentrifuge tubes filled with a buffer consisting of 0.65 M sucrose, 25 mM MES pH 5.7, 10 mM NaCl, 5 mM CaCl_2_ and 0.03% (w/v) of α-DDM (sucrose gradient buffer). For the crosslinking experiment with glutaraldehyde, 0.1% (v/v) glutaraldehyde was added to the sucrose gradient buffer. Centrifugation was carried out at 100,000 *g* for 12 h at 4 °C (Surespin 630 rotor, Thermo Scientific). The sucrose band containing PSII-LHCII supercomplexes was harvested and, if necessary, concentrated to a Chl concentration above 1.25 mg mL^−1^ by membrane filtration with an Amicon Ultra 100 kDa cutoff device (Millipore) and then stored at −80 °C. Remaining glutaraldehyde was inactivated after fractionation by adding Tris-HCl pH 8.0 to a final concentration of 80 mM.

### Spectroscopic analyses

Absorption spectra in native conditions were recorded using a Lambda25 spectrophotometer (Perkin Elmer) at 12 °C.

### Gel electrophoresis

Separation of thylakoid membrane protein complexes and PSII-LHCII complexes was performed using large pore blue native polyacrylamide gel electrophoresis^57^ (lpBN-PAGE), adopting the protocol previously described^48^.

### Mass spectrometry analyses

PSII-LHCII supercomplexes were centrifuged at 20,000 *g* for 10 min at 4 °C, and the resulting pellet was rinsed in 10 mM HEPES pH 7.5. To remove the adhered pigments, proteins were precipitated in ice-cold acetone overnight at −20 °C. The extracting solution was centrifuged at 20,000 *g* for 20 min at 4 °C, and the denatured proteins were subsequently re-solubilized in a buffer consisting of 50 mM Tris-HCl pH 8.0, 7 M urea and 2 M thiourea. Insoluble material was removed by centrifuging at 15,000 *g* for 10 min. Protein concentration was determined using the Bradford assay^58^. Proteins at a concentration of 0.5 mg mL^−1^ were reduced with 10 mM DTT for 30 min at 37 °C and alkylated with 20 mM iodoacetamide for 30 min at room temperature in the dark. The protein in-solution digestion was conducted by adding Trypsin/Lys-C Mix (Promega, WI, USA) at a final protein:protease ratio of 25:1 (w/w), followed by overnight incubation at 37 °C. Peptide desalting was conducted by solid phase extraction^59^ using 30 mg Oasis HLB cartridges (Waters, MA, USA). Peptides were dried and then dissolved in 30 µl of LC-MS/MS mobile phase A (water containing 0.1% (v/v) formic acid).

LC-MS/MS analyses were performed using a micro-LC Eksigent Technologies (Dublin, USA) system, with a Halo Fused C18 column (0.5 × 100 mm, 2.7 μm; Eksigent Technologies Dublin, USA) as the stationary phase. The mobile phase was a mixture of 0.1% (v/v) formic acid in water (A) and 0.1% (v/v) formic acid in acetonitrile (B), eluting at a flow-rate of 15.0 μL min^−1^ and at an increasing concentration of solvent B from 2% to 40% over 30 min. The injection volume was 4.0 μL. The LC system was interfaced with a 5600 + TripleTOF^TM^ system (AB Sciex, Concord, Canada) equipped with DuoSpray^TM^ Ion Source and CDS (Calibrant Delivery System). The mass spectrometer was operated in information dependent acquisition (IDA) mode. Peptide profiling was performed using a mass range of 100–1600 Da (TOF scan with an accumulation time of 100.0 ms), followed by a MS/MS product ion scan from 200 to 1250 Da (accumulation time of 5.0 ms) with the abundance threshold set at 30 cps. The ion source parameters in electrospray positive mode were set as follows: curtain gas (N_2_) at 25 psig, nebulizer gas GAS1 at 25 psig, and GAS2 at 20 psig, ion spray floating voltage (ISFV) at 5000 V, source temperature at 450 °C and declustering potential at 25 V.

MS data were acquired with Analyst TF 1.7 (AB SCIEX, Concord, Canada). Raw files were processed with the search engine ProteinPilot^TM^ v.5.0.1.0, 4895 (AB Sciex, Concord, Canada) using the Paragon algorithm v.5.0.1.0, 4874. The following sample parameters were used: Trypsin/Lys-C digestion, cysteine alkylation set to carbamidomethylation and no special factors. Processing parameters were set to “Biological modification”. All data files were searched, thorough ID search effort, using a UniProtKB/TrEMBL database containing Viridiplantae proteins (version 2016.09.02, with a total of 3,825,803 sequences), or a *de novo* database obtained by generating open reading frames from all six frames of each transcript in the *P*. *sativum* transcriptome (p.sativum_csfl_reftransV1 downloaded from https://www.coolseasonfoodlegume.org/organism/Pisum/sativum/reftrans/v1) concatenated with a reversed “decoy” version of the “forward” database. After searching, we accepted protein IDs that had a ProteinPilot Unused Score of at least 1.3 (equivalent to a 95% confidence interval) as a cutoff threshold and an estimated local false discovery rate (FDR) not higher than 1% according to Rardin *et al*.^60^.

### Sequence analysis

Clustal Omega^61^ and Jalview^62^ software were used for multiple sequence alignment and conservation analysis, respectively. A phylogenetic tree was generated with ClustalW2.0 software^63^ using the UPGMA method and PAM250 substitution matrix.

### Cryo-EM data collection

PSII-LHCII supercomplexes were prepared at 1 mg mL^−1^ in the sucrose gradient buffer. 4 μL of sample were applied to a glow-discharged lacey carbon grid (200 Cu mesh, Quantifoil) within the chamber of a Vitrobot (mark 3, FEI). After 60 s incubation at 100% humidity and 21 °C, a quick wash with 4 μL of 10 mM Hepes pH 7.5 was performed to remove sugar, excess solution was blotted from both sides for 4 s, and the grid was plunge-frozen in a liquid ethane/propane mixture. Samples were exposed to only dim green light during the grid preparation procedure. Data collection was performed on a Titan Krios microscope (FEI) operated at 300 kV using EPU automated acquisition software (FEI). Spanning a defocus range −1 to −3 μm, 6,834 micrographs were recorded on a Falcon II direct electron detector (FEI) at 59,000 magnification (image pixel size of 1.4 Å), with a total dose of 47.5 e^−^/Å^2^ fractionated over 7 frames (1.5 s exposure, dose rate of 30 e^−^/Å^2^/s).

### Data processing and 3D reconstruction

Beam-induced motion was corrected by aligning the image frames with in-house developed software (https://github.com/dtegunov/k2align). Aligned images were used for single particle analysis. All image processing steps were performed using the Scipion platform (http://scipion.cnb.csic.es), which is an image processing framework that integrates several software packages into a unified interface^64^. For estimating the objective lens defocus parameters (i.e., contrast transfer function/CTF) in the transmission electron micrographs, we used the CTFFIND4 program^65^. For this project, a total of 33,729 cryo-EM particles were manually extracted from 6,834 micrographs using the particle picking tool of Xmipp^66^. These particles were classified in 2D using Xmipp^67^ and then in 3D using Relion^68^. Using e2initialmodel.py of EMAN2.1^69^ and Ransac of Xmipp^70^, averages assigned to the PSII-LHCII supercomplex were used to generate several initial models and subsequently select among them the one with the highest score to be used as an unbiased low-resolution 3D template for refinement and classification. This template was used as an initial model for Relion 3D classification^68^. After 3D classification, 14,291 particles were identified as paired C_2_S_2_M supercomplexes, 7,021 particles as paired C_2_S_2_ supercomplexes and 5,641 particles as unpaired C_2_S_2_M supercomplexes. The paired C_2_S_2_M particles were processed further with 3D refinement, using the Relion auto-refine algorithm^68^ and D2 point group symmetry. After refinement, we used the multireference alignability method, recently implemented in Scipion, to select the “best particles” in the dataset according to the reference map and provide the alignment accuracy for each particle used in the reconstruction^71^. The final cryo-EM model was validated using the soft-alignment validation approach recently described by Vargas *et al*.^71^.

### Fitting of atomic models into cryo-EM maps

UCSF Chimera^72^ software was used to model atomic structures into the 3D cryo-EM reconstruction. Surface-rendered views of the EM density were calculated at a threshold of 2.5 σ. Atomic co-ordinates from spinach^26^ (PDB ID: 3JCU) were used for modeling both the central dimeric PSII core (removing chains P, Q and U, corresponding to the extrinsic subunits PsbP, PsbQ and PsbTn, respectively) and the monomeric Lhcb4 and Lhcb5 subunits. Atomic coordinates from pea^36^ (PDB ID: 2BHW) were used for fitting the LHCII S- and M-trimers. For the monomeric Lhcb6, we fitted the 3D structure predicted by the PHYRE2 algorithm^39^ for the *P*. *sativum* Lhcb6 protein sequence derived from the corresponding transcript (p.sativum_csfl_reftransV1_0079196_5/148-357). Local fitting and adjustment of the Lhcb6 subunit and the LHCII trimers in the cryo-EM maps were performed by visual inspection, aided in the case of the S-trimer by its known localization in the available C_2_S_2_ atomic structure^26^.

### Negative stain EM

PSII-LHCII supercomplexes were prepared at 1 mg mL^−1^ in the sucrose gradient buffer. To remove salts from the sample in a centrifugation step, we used Amicon Ultra 100 kDa cutoff buffer exchange spin columns (Millipore) and a salt-free buffer containing 25 mM MES pH 5.7 and 0.03% (w/v) α-DDM at a final ratio 1:60 (v/v). Concentrated samples were diluted to a final Chl concentration of ~30 µg mL^−1^ either in the sucrose gradient buffer or in the buffer with no salts. Samples were applied to glow discharged carbon-coated copper grids, washed quickly with distilled water and negatively stained with 2% (w/v) uranyl acetate. A FEI Tecnai F20-ST transmission electron microscope, equipped with a field emission gun (FEG) operated at 200 kV, was used for acquisition of micrographs, recorded at 38,000 magnification on a Gatan Orius 4.0 K × 2.7 K CCD camera.

### Chlorophyll *a* fluorescence induction measurements

PSII-LHCII supercomplexes prepared at 1.25 mg mL^−1^ in the sucrose gradient buffer were used for the experiments. Samples were diluted to a final Chl concentration of 1.25 μg mL^−1^ in the sucrose gradient buffer or in a similar buffer devoid of any salt. The fluorescence induction OJIP transient was measured at room temperature, with a FL3500 double modulation fluorometer (Photon Systems Instruments). Dark-adapted samples were illuminated for 1 s with continuous actinic light (2,400 μmol photons m^−2^ s^−1^, emission peak at 630 nm). The first reliable point of the transient is measured at t_0_ = 0.02 ms after the onset of illumination. After recording fast fluorescence transients, the OJIP curves were analyzed according to methods previously reported^49–53^ to estimate the energetic connectivity between PSII units. Results shown in graphs and tables are presented as the mean value ± standard deviation of six replicates.

### Molecular dynamics simulations

The atomic model generated by fitting the 3D cryo-EM map of the paired C_2_S_2_M supercomplexes (Fig. 2) was embedded within a pair of thylakoid membranes constituted by phosphatidylglycerol (PG), digalactosyldiacylglycerol (DGDG), monogalactosyldiacylglycerol (MGDG) and sulfoquinovosyl-diacylglycerol (SQDG) molecules, according to the detailed thylakoid membrane composition of higher plants proposed by van Eerden *et al*.^73^. The starting structure for the molecular dynamics simulations was the atomic coordinates of the protein components modeled into the cryo-EM 3D map, with the exclusion of any bound cofactors, and the predicted structure of Lhcb6. Prior to the insertion of the paired PSII-LHCII supercomplexes into the pair of membranes, the supercomplexes and the membranes were separately energy minimized and equilibrated. Subsequently, the supercomplex pair was embedded into the thylakoid membranes and solvated with water molecules and Na^+^ and Cl^−^ ions according to van Eerden *et al*.^73^ in order to reach a globally uncharged system.

A 7 ns fine-grained simulation was performed with the GROMACS 4.6.6 package^74^, with the system interactions modeled by the GROMOS G53a6 force field^75^. The system was simulated using the Nosé-Hoover thermostat to control the temperature^76, 77^. By setting the thermal bath temperature at 300 K, the paired PSII-LHCII supercomplex, the pair of thylakoid membranes and the solvent were independently coupled every 0.5 ps. The pressure was semi-isotropically coupled every 2 ps to a reference pressure of 1 bar using the Parrinello-Rahman barostat^78^, and a compressibility of 4.6 × 10^−5^ bar^−1^ was applied to the system. Electrostatic and Van der Waals interactions were calculated using the Particle Mesh Ewald potential with a 1.4 nm cutoff. The mobility of the PSII-LHCII supercomplex pair was determined by calculating the root mean square fluctuations (RMSF) of backbone atoms (N, Cα and C atoms) during the molecular dynamics simulations with respect to the reference time-averaged structure:

$$RMS{F}_{i}=\sqrt{\langle {({r}_{i}-\langle {r}_{i}\rangle )}^{2}\rangle }$$

where ${r}_{i}$ is the i^−th^ backbone atoms and $\langle \rangle$ denotes the time-average.

### Data availability

The data that support the findings of this study are available from the corresponding author upon request. The cryo-EM map was deposited at the Electron Microscopy Data Bank (EMDB) with accession code EMD-3825.

### Addendum

After the completion of this manuscript, a paper was published by van Bezouwen *et al*.^79^, which reported a 5.3 Å structure of the higher plant unpaired C_2_S_2_M_2_ supercomplex. This PSII-LHCII supercomplex was isolated from *Arabidopsis* and its structure determined by single particle cryo-EM. In this work, the supramolecular organization of the subunits in the C_2_S_2_M_2_ is described in particular with respect to the location and orientation of trimeric and monomeric LHCII, and the location of all the chlorophylls in the subunits and their role in the energy flow from the peripheral light harvesting complexes to the PSII core is discussed. It is worth noting that, despite the lower resolution of our cryo-EM map, the fitting of the C_2_S_2_M components, and in particular of the LHCII trimers and the Lhcb6 subunit, is perfectly in accordance with that proposed by van Bezouwen *et al*.^79^. It is also worth noting that most of the chlorophylls responsible for energy connections observed in this recent structure are on the stromal side of the LHCII complexes. This evidence may suggest their involvement in the energy exchange between PSII cores located in adjacent thylakoid membranes *in vivo*, in accordance to the increase of PSII connectivity measured in our isolated paired PSII-LHCII supercomplexes.

## Electronic supplementary material

Supplementary information

Supplementary video
