# Supplementary material for: Pea PSII-LHCII supercomplexes form pairs by making connections across the stromal gap
Source: Sci Rep. 2017 Aug 30;7:10067. doi: 10.1038/s41598-017-10700-8 (PMC5577252; doi:10.1038/s41598-017-10700-8)
Supplement: Supplementary file 1 — Supplementary information [file 41598_2017_10700_MOESM1_ESM.pdf]

Pea PSII-LHCII supercomplexes form pairs by making connections across the stromal gap

Pascal Albanese[a,b], Roberto Melero[c], Benjamin D Engel[d], Alessandro Grinzato[e], Paola Berto[e], Marcello Manfredi[f,g], Angelica Chiodoni[h], Javier Vargas[c], Carlos Óscar Sánchez Sorzano[c], Emilio Marengo[g], Guido Saracco[h], Giuseppe Zanotti[e], Jose-Maria Carazo[c], Cristina Pagliano[a],*

SUPPLEMENTARY INFORMATIONS

[a]Applied Science and Technology Department–BioSolar Lab, Politecnico di Torino, Viale T. Michel 5, 15121 Alessandria, Italy
[b]Department of Biology, University of Padova, Via Ugo Bassi 58 B, 35121 Padova, Italy
[c]Biocomputing Unit, Centro Nacional de Biotecnología–CSIC, Darwin 3, Cantoblanco, 28049, Madrid, Spain
[d]Department of Molecular Structural Biology, Max Planck Institute of Biochemistry, 82152 Martinsried, Germany
[e]Department of Biomedical Sciences, University of Padova, Via Ugo Bassi 58 B, 35121 Padova, Italy
[f]ISALIT–Department of Science and Technological Innovation, University of Eastern Piedmont, Viale T. Michel 11, 15121 Alessandria, Italy
[g]Department of Science and Technological Innovation, University of Eastern Piedmont, Viale T. Michel 11, 15121 Alessandria, Italy
[h]Center for Sustainable Future Technologies – CSFT@POLITO, Istituto Italiano di Tecnologia, Corso Trento 21, 10129 Torino, Italy

Corresponding author*
Cristina Pagliano E-mail: cristina.pagliano@polito.it; Tel. No. +39 131 229301; Fax No. +39 131 229344; Politecnico di Torino, Applied Science and Technology Department - BioSolar Lab, Viale T. Michel 5, 15121 Alessandria, Italy

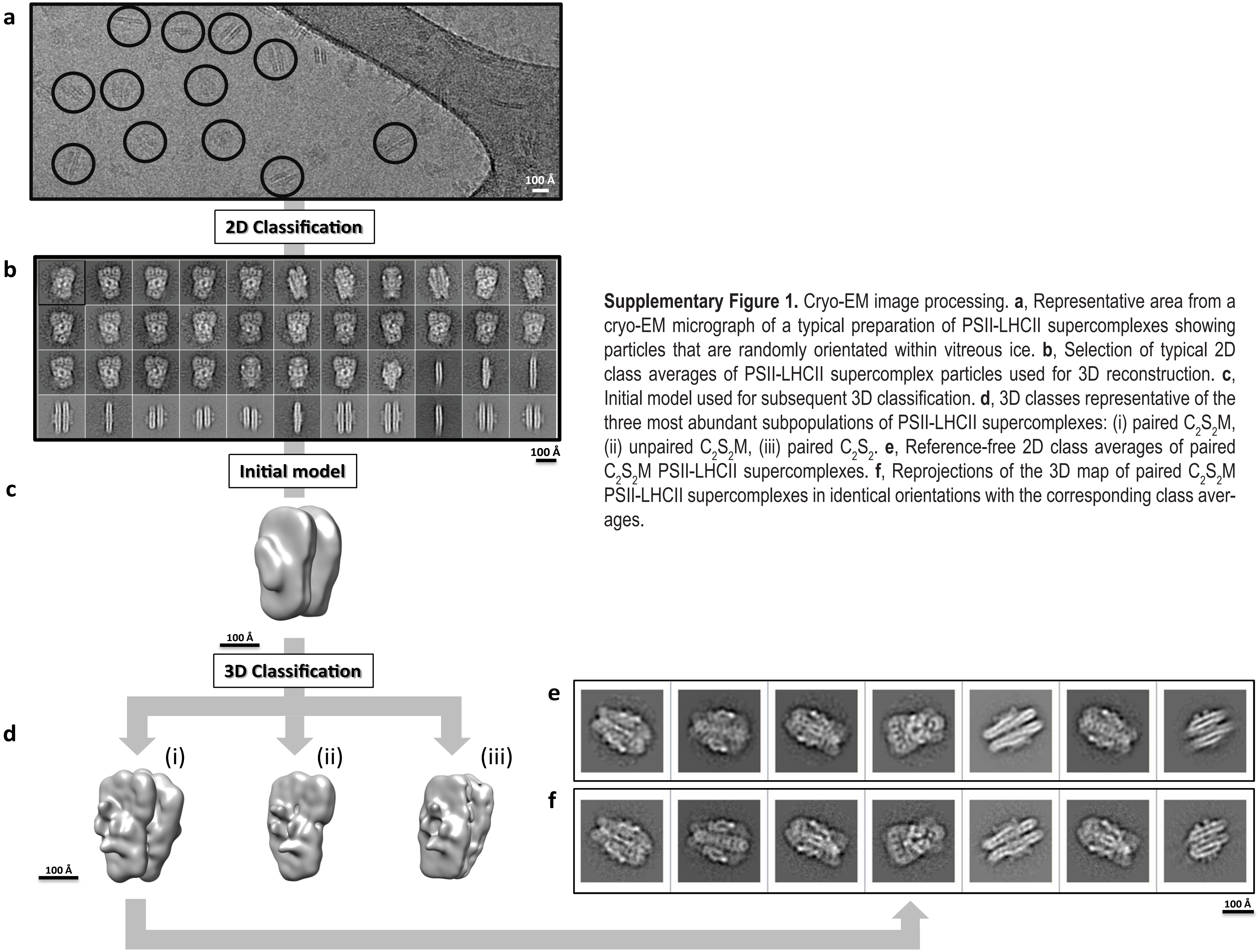

Supplementary Figure 1. Cryo-EM image processing. **a**, Representative area from a cryo-EM micrograph of a typical preparation of PSII-LHCII supercomplexes showing particles that are randomly orientated within vitreous ice. **b**, Selection of typical 2D class averages of PSII-LHCII supercomplex particles used for 3D reconstruction. **c**, Initial model used for subsequent 3D classification. **d**, 3D classes representative of the three most abundant subpopulations of PSII-LHCII supercomplexes: (i) paired C_2S_2M, (ii) unpaired C_2S_2M, (iii) paired C_2S_2. **e**, Reference-free 2D class averages of paired C_2S_2M PSII-LHCII supercomplexes. **f**, Reprojections of the 3D map of paired C_2S_2M PSII-LHCII supercomplexes in identical orientations with the corresponding class averages.

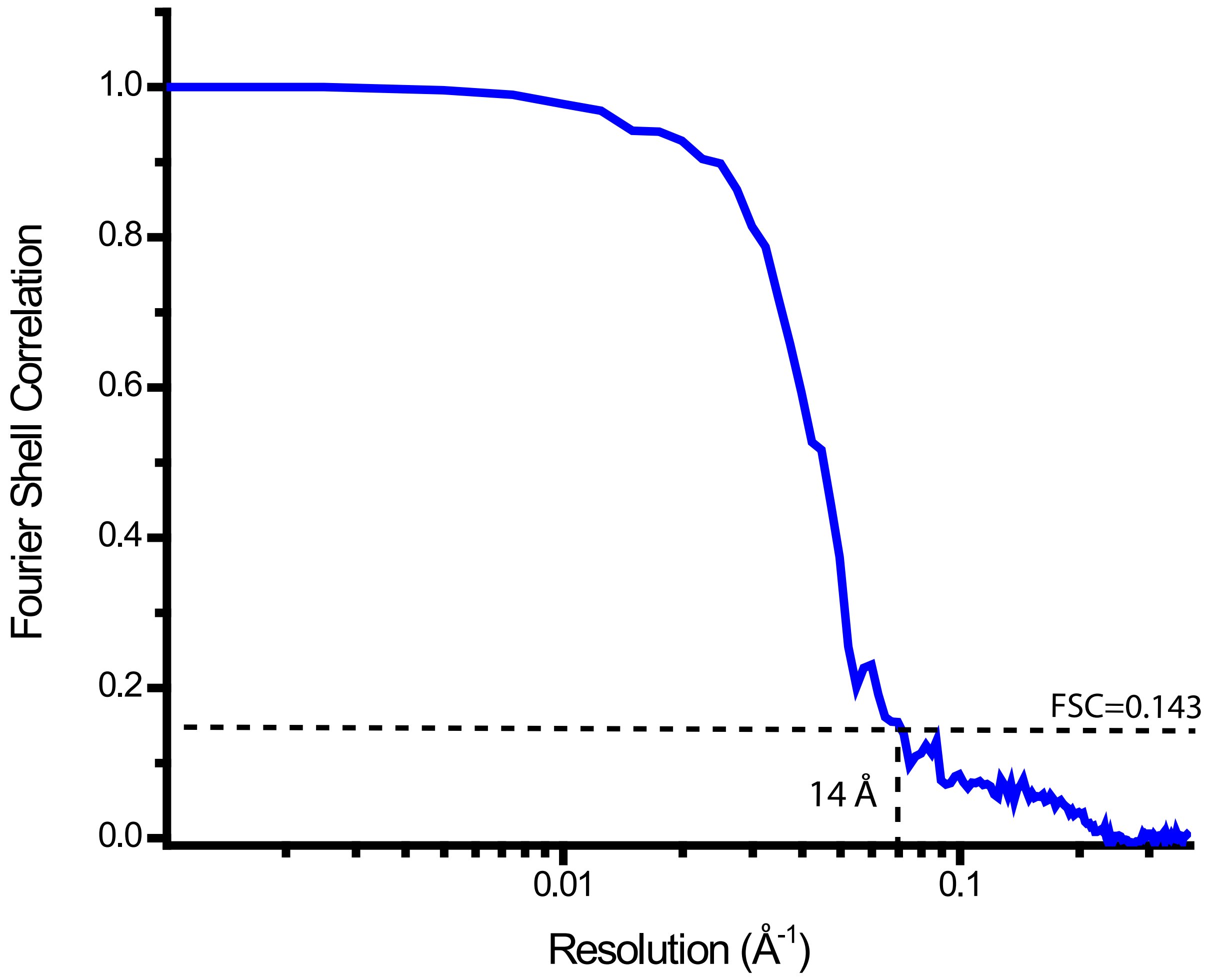

Supplementary Figure 2. Evaluation of the resolution of the cryo-EM structure of paired C_2S_2M PSII–LHCII supercomplexes. Gold standard Fourier Shell Correlation (FSC) curve of the cryo-EM reconstruction of paired C_2S_2M PSII–LHCII supercomplexes. The resolution estimate is 14 Å at the 0.143 FSC cutoff criterion.

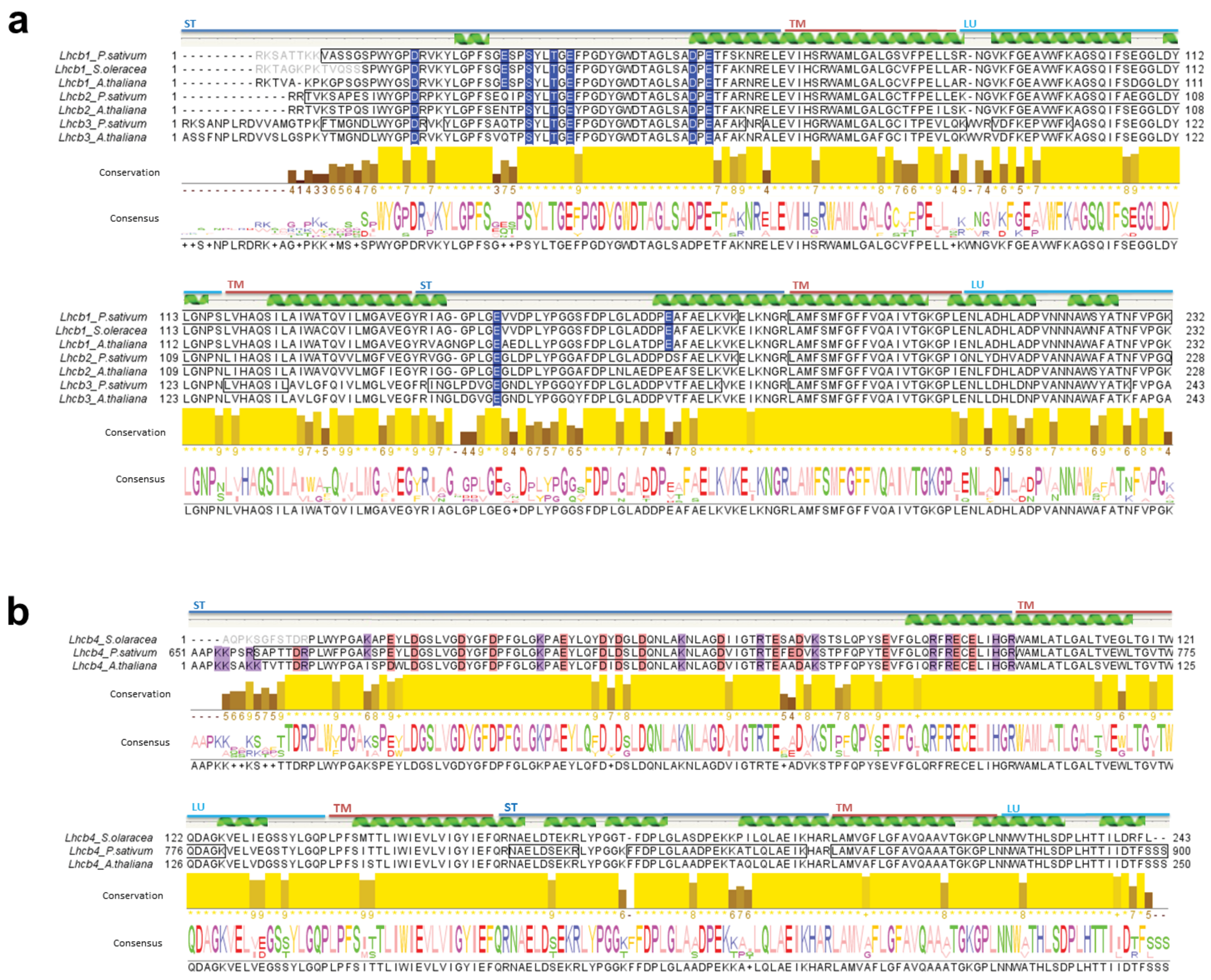

Supplementary Figure 3. Sequence comparison of Lhcb1-3 and Lhcb4 from different plant species. a, Multiple sequence alignment of UniProtKB/TrEMBL mature amino acid sequences of Lhcb1 from *P. sativum* (sp|P07371|CB22_PEA; corresponding PDB 2BHW, residues without coordinates in grey), *S. oleracea* (sp|P12333|CB2A_SPIOL; corresponding PDBs 4LCZ, 1RWT, 3JCU, residues without coordinates in grey) and *A. thaliana* (sp|P0CJ48|CB1A_ARATH), together with Lhcb2 and Lhcb3 from *P. sativum* (sp|P27520|CB215_PEA and tr|Q5I8X1|Q5I8X1_PEA, respectively) and *A. thaliana* (tr|Q9SYW9|Q9SYW9_ARATH and sp|Q9S7M0|Q9S7M0_ARATH, respectively). Residues involved in grana stacking according to Wan *et al.*, (2014) (PDB 4LCZ) are highlighted with a blue background. **b**, Multiple sequence alignment of mature amino acid sequences of Lhcb4.1-4.2 proteins from *S. oleracea* (tr|F2Z293|F2Z293_SPIOL; corresponding PDBs 3JCU:R, 3PL9, residues without coordinates in grey), *P. sativum* (deduced from transcriptome reftransV1_0076852_5/651-900) and *A. thaliana* (sp|Q07473|CB4A_ARATH, Lhcb4.1). Charged amino acid residues at the N-terminus are highlighted with red (negative) and purple (positive) backgrounds. Partial amino acid sequences of Lhcb1-4 in *P. sativum* identified by mass spectrometry analyses are highlighted in black boxes (see Supplementary Table 1). Secondary structure with alpha-helix in green is based on PDBs 2BHW for Lhcb1 (**a**) and 3JCU:R for Lhcb4 (**b**); topology of the Lhcb subunits with respect to the thylakoid membrane (transmembrane, TM; luminal, LU; stromal, ST) is based on reviewed UniProtKB entries for Lhcb1 (sp|P07371|CB22_PEA) and Lhcb4.1 (sp|Q07473|CB4A_ARATH).

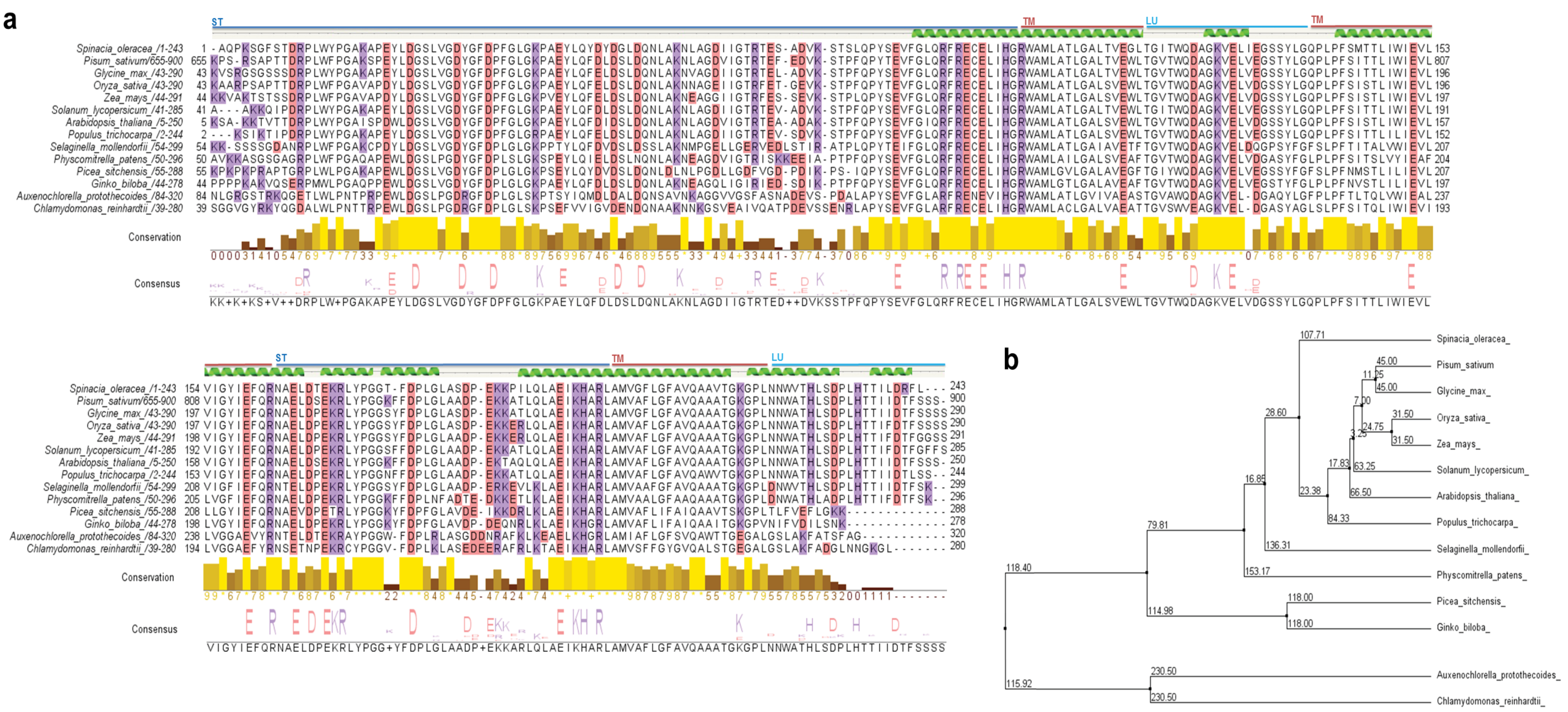

Supplementary Figure 4. Sequence comparison of Lhcb4 from different species and phylogeny reconstruction. a,**b**, Multiple sequence alignment (**a**) and phylogenetic tree (**b**) of mature amino acid sequences of Lhcb4.1-4.2 proteins from various organisms of the Viridiplantae lineage. There are two green algae serving as an out-group (*C. reinhardtii* sp|Q93WD2|CB29_CHLRE; *A. protothecoides* tr|A0A087SNX4|A0A087SNX4_AUXPR), in addition to two gymnosperms (*G. biloba* tr|S4X0Q5|S4X0Q5_GINBI; *P. sitchensis* tr|A9NKX-0|A9NKX0_PICSI) and two basal land-plants (the moss *P. patens* tr|A9T2F8|A9T2F8_PHYPA; the lycophyte *S. moellendorffii* tr|D8RTB9|D8RTB9_SELML). Within angiosperm lineage, there are two monocotyledons (*O. sativa* tr|O65217|O65217_ORYSA; *Z. mays* tr|O24561|O24561_MAIZE) and six eudicotyledons (*P. sativum* >reftransV1_0076852_5/651-900; *A. thaliana* sp|Q07473|CB4A_ARATH; *G. max* tr|I1J7A8|I1J7A8_SOYBN; *P. trichocarpa* tr|B9IG87|B9IG87_POPTR; *S. lycopersicum* tr|K4CRS9|K4CRS9_SOLLC and *S. oleracea* tr|F2Z293|F2Z293_SPIOL). Secondary structure with alpha-helix in green is based on PDB 3JCU:R (*S. oleracea* tr|F2Z293|F2Z293_SPIOL). Topology with respect to the thylakoid membrane (transmembrane, TM; luminal, LU; stromal, ST) is based on reviewed UniProtKB Lhcb4.1 (*A. thaliana* sp|Q07473|CB4A_ARATH). Charged amino acid residues are highlighted with red (negative) and purple (positive) background.

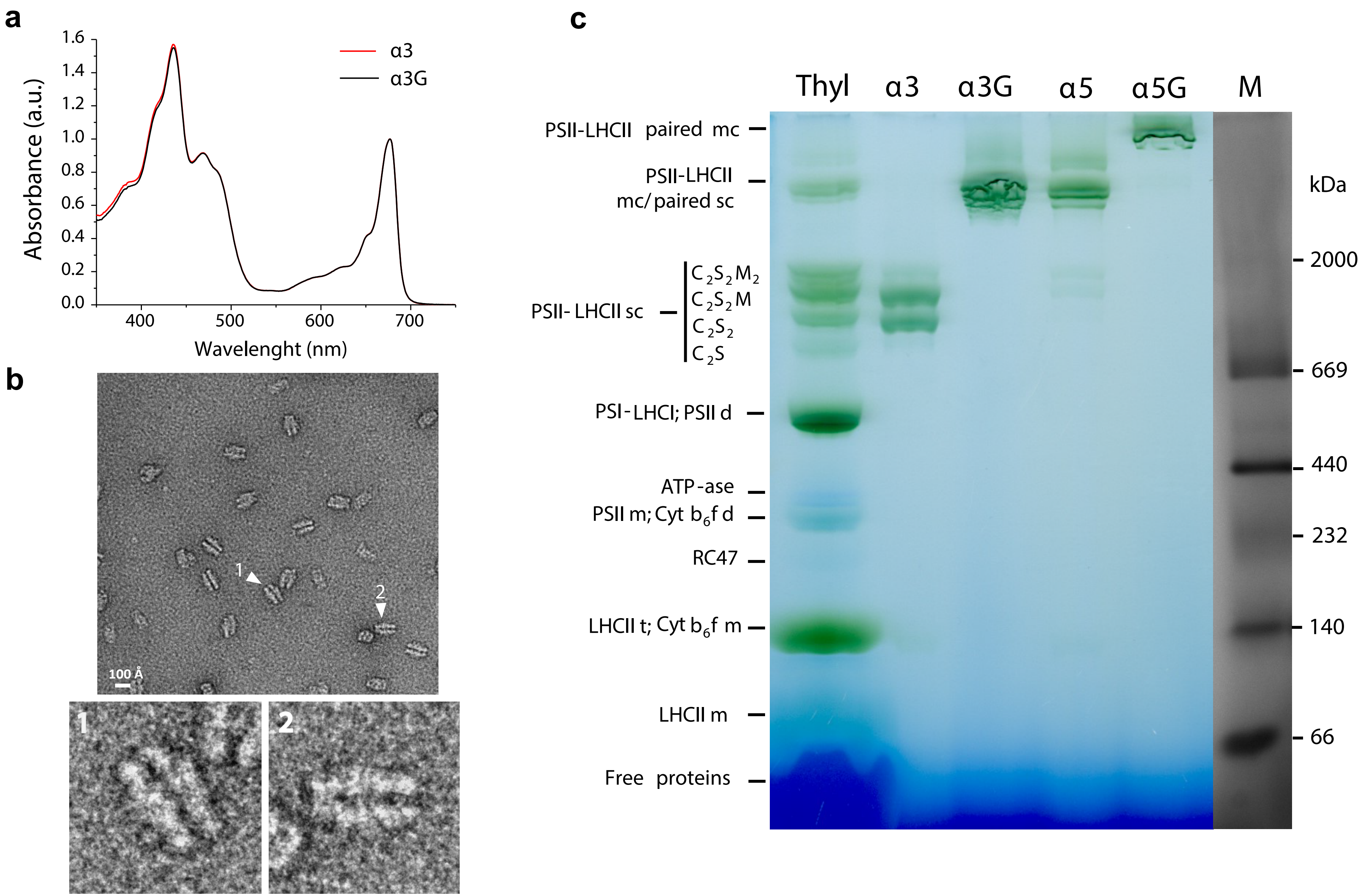

Supplementary Figure 5. Characterization of pea PSII-LHCII supercomplexes isolated in the presence or absence of glutaraldehyde. **a**, Absorption spectra, normalized to the maximum in the red region, obtained from sucrose gradient bands α3 and α3G, which contain the PSII-LHCII supercomplexes shown in Fig. 4a. **b**, Electron micrograph of particles contained in sucrose gradient band α3G, negatively stained with 2% uranyl acetate, showing possible stromal connections between the paired supercomplexes (see zoom 1 and 2 of the particles indicated with white triangles in the figure). **c**, lpBN-PAGE of thylakoid membranes (25 µg Chl) and sucrose gradient bands α3 and α3G (8 µg Chl). For comparison, bands α5 and α5G, which contain PSII-LHCII megacomplexes shown in Fig. 4a, were also loaded. Lane M is a mixture of native high molecular weight marker (GE Healthcare) and blue dextran (Sigma-Aldrich). Labels on the left indicate the main protein complexes of the solubilized thylakoid membranes, indexed as follows: megacomplex (mc), supercomplex (sc), trimer (t), dimer (d), monomer (m).

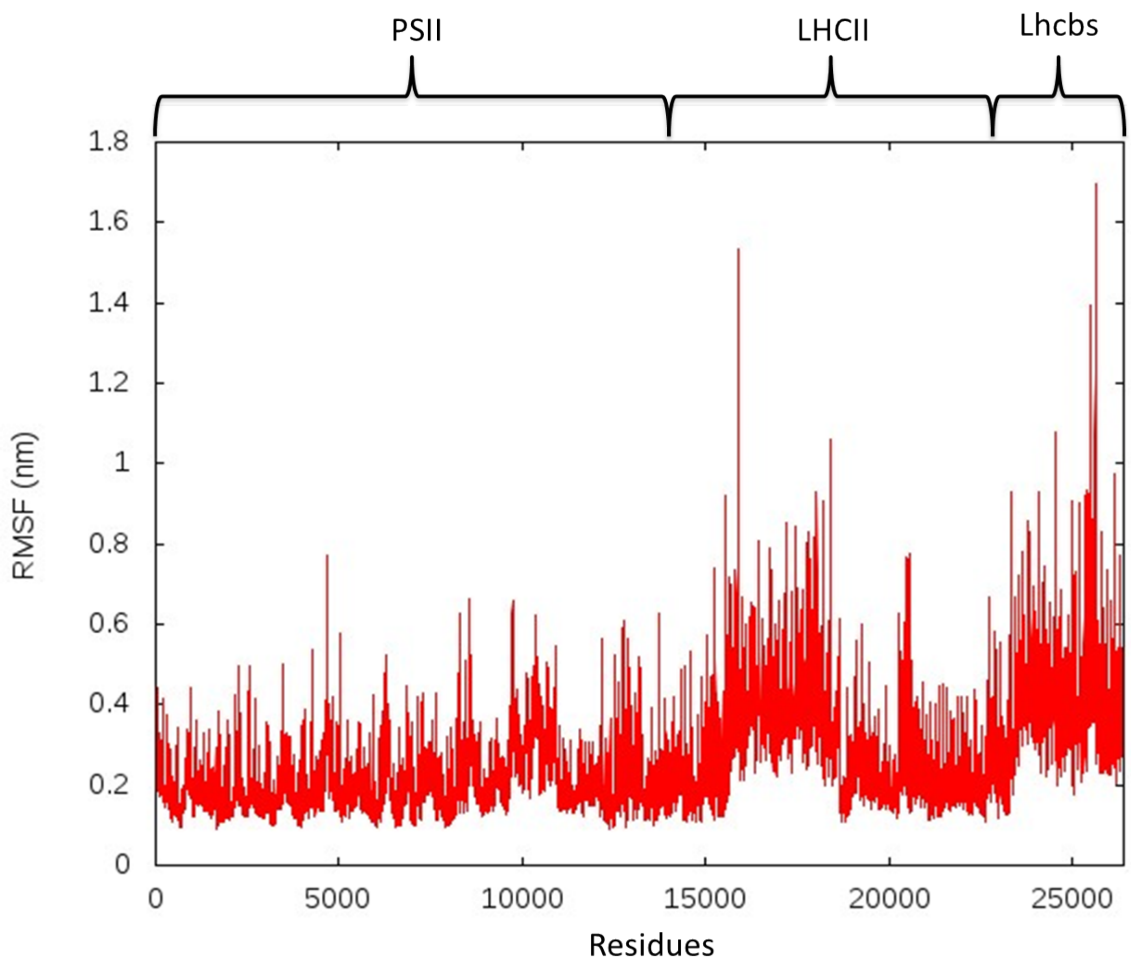

Supplementary Figure 6. Molecular dynamics simulations. Root mean square fluctuations (RMSF) for the backbone atoms of the paired C_2S_2M PSII-LHCII supercomplexes, evaluated for each residue at the end of the 7 ns molecular dynamics simulations. Residues are subdivided into the PSII core (PSII), the peripheral LHCII trimers (LHCII) and the monomeric Lhcb subunits (Lhcbs).

Supplementary Video. Molecular dynamics simulations of the paired C_2S_2M PSII-LHCII supercomplexes over a 7 ns timespan. Structures used for each protein component and coloring as in Fig. 2.

Supplementary Table 1. List of LHCII proteins identified by LC-MS/MS that are present in the PSII-LHCII supercomplex preparation used for cryo-EM. For each identified protein, the table reports: the protein name (first column), the calculated molecular weight (MW, second column), the unused score (third column), selected sequences of peptides with confidence >99% (fourth column), eventual modifications and cleavages (fifth column), the corresponding precursor ion mass (sixth column), the accession number of the protein (and reference organism) in the UniProtKB/TrEMBL database or accession number of the transcript used to derive the protein sequence (seventh column), the sequence coverage (eighth column), the percentage of identity between the sequence of the reference organism and that of *P. sativum* or *A. thaliana* (ninth column) and the percentage of identity between the sequence of the reference organism and that of *S. oleracea* (tenth column).

Protein	MW (Da)	Unused score	Peptide sequence (confidence >99%)	Modification/cleavage	Precursor ion mass (*m/z*)	UniProtKB/transcriptome accession (reference organism)	Sequence coverage	% Identity with *P. sativum* or *A. thaliana*	% Identity with *S. oleracea*
**Lhcb1**	28635	80.5	VASSGSPWYGPDRVK	Missed R-V@13	1604,795410	sp|P07371|CB22_PEA (*Pisum sativum*)	82%	100% sp|P07371|CB22_PEA *P. sativum*	89% sp|P12333|CB2A_SPIOL
			FGEAVWFK	Carbamyl@N-term	1025,496216				
			GLADDPEAFAELK	Cleaved L-G@N-term	1374,670898				
			GPLENLADHLSDPVNNNAWSYATNFVPGK		3139,499023				
			IAGGPLGEVVDPLYPGGSFDPLGLADDPEAFAELKVK	Missed K-V@35	3752,903809				
			WAMLGALGCVFPELLSR	Carbamidomethyl(C)@9	1918,976440				
			NRELEVIHSR	Missed R-E@2	1251,671753				
			LAMFSMFGFFVQAIVTGK	Oxidation(M)@3	2008,989502				
			YLGPFSGESPSYLTGEFPGDYGWDTAGLSADPETFSK		3944,759033				
			AGSQIFSEGGLDYLGNPSLVHAQSILAIWATQVILMGAVEGYR		4531,318848				
**Lhcb2**	28866	22.1	AGSQIFAEGGLDYLGNPNLIHAQSILAIWATQVVLMGFVEGYR		4619,350586	sp|P27520|CB215_PEA (*Pisum sativum*)	82%	100% sp|P27520|CB215_PEA *P. sativum*	—
			FGEAVWFK	Carbamyl@N-term	1025,496216				
			GPIQNLYDHVADPVANNAWAFATNFVPGQ		3125,495117				
			NRELEVIHSR	Missed R-E@2	1251,671753				
			VGGGPLGEGLDPLYPGGAFDPLGLADDPDSFAELK	Carbamidomethyl(D)@20	3512,648193				
			SAPESIWYGPDRPK		1601,782837				
			WAMLGALGCTFPELLEK	Carbamidomethyl(C)@9	1934,964478				
			YLGPFSEQIPSYLTGEFPGDYGWDTAGLSADPETFAR	Cation:K(E)@16	4091,822998				
**Lhcb3**	28710	22.1	INGLPDVGEGNDLYPGGQYFDPLGLADDPVTFAELK		3805,833252	tr|Q5I8X1|Q5I8X1_PEA (*Pisum sativum*)	58%	100% tr|Q5I8X1|Q5I8X1_PEA *P. sativum*	—
			GPLENLLDHLDNPVANNAWVYATK	Cleaved A-G@N-term	2663,334473				
			YLGPFSAQTPSYLTGEFPGDYGWDTAGLSADPEAFAK	Cleaved M-G@N-term	3925,792236				
			LAMFSMFGFFVQAIVTGK		1993,026978				
			VDFKEPVWFK	Carbamyl@N-term; Missed K-E@4	1336,678101				
			WAMLGALGCITPEVLQK	Carbamidomethyl(C)@9	1886,017822				
			FTMGNDLWYGPDR		1570,693115				
**Lhcb4.1/4.2**	27481	34.4	ATLQLAEIK		985,5804443	p.sativum_csfl_reftransV1_0076852_5/651-900 (*Pisum sativum*)	80%	92% sp|Q07473|CB4A_ARATH *A. thaliana* (Lhcb4.1)<br>92% sp|Q9XF88|CB4B_ARATH *A. thaliana* (Lhcb4.2)	86% tr|F2Z293|F2Z293_SPIOL
			FFDPLGLAADPEKK	missed K-K@13	1546,803711				
			FRECELIHGR	missed R-E@2; Carbamidomethyl(C)@4	1315,644165				
			LAMVAFLGFAVQAAATGK		1764,957031				
			NAELDSEKR	missed K-R@8	1060,513916				
			NLAGDVIGTR		1014,546265				
			SPEYLDGSLVGDYGFDPFGLGKPAEYLQFDLDSLDQNLAK		4393,094727				
			STPFQPYTEVFGLQR		1768,877686				
			WAMLATLGALTVEWLTGVTWQDAGK		2717,363037				
			TEFEDVK		866,4006958				
			SAPTTDRPLWFPGAK	Carbamyl@N-term	1685,856323				
			GPLNNWATHLSDPLHTTIIDTFSSS	cleaved S-F@C-term	2723,321289				
**Lhcb4.3**	27537	11.6	FFDPLGLANDPEEKER	missed K-E@14	1875,900269	p.sativum_csfl_reftransV1_0068262_4/95-343 (*Pisum sativum*)	55%	75% sp|Q9S7W1|CB4C_ARATH *A. thaliana*	75% tr|A0A0K9RW58|A0A0K9RW58_SPIOL
			GFDPLGFAKPAEYLQFDLDSLDQNLAK		3011,480225				
			VEAGEVKPTPFQPYSEVFGIER		2478,242188				
			VRQPESDGLVWFPGAQPPEWLDGTMIGDR	missed R-Q@2	3252,578125				
**Lhcb5**	25322	33.8	IFLPDGLLDRSEIPEYLTGEVPGDYGYDPFGLSK	missed R-S@10	3771,856934	p.sativum_csfl_reftransV1_0087274_3/136-367 (*Pisum sativum*)	71%	89% sp|Q9XF89|CB5_ARATH *A. thaliana*	86% tr|A0A0K9QUQ7|A0A0K9QUQ7_SPIOL
			ITNGLDLEDKFHPGGPFDPLGLANDPDQAAILK	missed K-F@10; Deamidated(N)@3	3488,74292				
			SEIPEYLTGEVPGDYGYDPFGLSKKPEDFAK	missed K-K@24	3447,6521				
			VVAPANEELAK		1139,618042				
			YGANCGPEAVWFK	Carbamidomethyl(C)@5	1497,671021				
			YQGYELIHAR		1248,625732				
			TGALLLDGGTLNYFGK	cleaved K-P@C-term	1638,851929				
**Lhcb6**	22841	13.7	FFDPLSLAGTIENGVYIPDTDK		2411,189453	p.sativum_csfl_reftransV1_0079196_5/148-357 (*Pisum sativum*)	50%	88% tr|Q9XF90|Q9XF90_ARATH *A. thaliana*	88% sp|P36494|CB4_SPIOL
			RWVDFFNPDSQSVEWATPWSK	missed R-W@1	2581,197266				
			SWIPGVSFGGNLVDPEWLDGSLPGDFGFDPLGLGKDPAFLK	missed K-D@35	4316,150879				
			TAENFVNSTGEQGYPGGK		1854,841553				

Supplementary Table 2. Definition of selected parameters derived from fast fluorescence kinetic measurements according to Strasser and Stirbet (2001)

$W_E = 1 - \left(\frac{F_{2ms} - F_{0.3ms}}{F_{2ms} - F_{0.05ms}}\right)^{1/5}$	Model-derived value of relative variable fluorescence at 100 µs calculated for unconnected PSII units
$W = \frac{F_{0.1ms} - F_{0.05ms}}{F_{2ms} - F_{0.05ms}}$	Relative variable fluorescence at 100 µs
$V_J = \frac{F_{2ms} - F_{0.05ms}}{F_M - F_{0.05ms}}$	
$C = \frac{W_E - W}{V_J W(1 - W_E)}$	Curvature constant of initial phase of the O–J curve
$p_{2G} = C \frac{F_{0.05ms}}{F_{2ms} - F_{0.05ms}}$	Overall grouping probability
$p = \frac{p_{2G}\left(\frac{F_M}{F_{0.05ms}} - 1\right)}{1 + p_{2G}\left(\frac{F_M}{F_{0.05ms}} - 1\right)}$	Connectivity parameter
$\boldsymbol{\omega} = p \frac{F_M - F_{0.05ms}}{F_M}$	Probability of the connectivity among PSII units

References

Strasser, R. J. & Stirbet, A. D. Estimation of the energetic connectivity of PS II centres in plants using the fluorescence rise O-J-I-P - Fitting of experimental data to three different PS II models. *Math. Comput. Simul.* 56, 451–461 (2001).

Wan T, Li M, Zhao X, Zhang J, Liu Z & Chang W. Crystal Structure of a Multilayer Packed Major Light-Harvesting Complex: Implications for Grana Stacking in Higher Plants. *Mol. Plant* **7**: 916–919 (2014).
